# Born to run? Associations between gestational and early‐life exposures and later‐life performance outcomes in Thoroughbreds

**DOI:** 10.1111/evj.70084

**Published:** 2025-08-25

**Authors:** Rebecca Mouncey, Amanda M. de Mestre, Juan Carlos Arango‐Sabogal, Kristien L. Verheyen

**Affiliations:** ^1^ Department of Pathobiology and Population Sciences Royal Veterinary College Hatfield UK; ^2^ Baker Institute for Animal Health, College of Veterinary Medicine Cornell University Ithaca New York USA; ^3^ Département de pathologie et microbiologie, Faculté de médecine vétérinaire Université de Montréal Quebec Canada

**Keywords:** exercise, foal, horse, performance, racing, Thoroughbred

## Abstract

**Background:**

Gestational and early‐life exposures may modulate development during growth and influence future athletic performance.

**Objectives:**

To investigate associations between gestational and early‐life exposures in Thoroughbreds and (i) likelihood of racing, (ii) total number of runs and (iii) total prizemoney by the end of the 3‐year‐old year.

**Study Design:**

Prospective cohort.

**Methods:**

Daily records were kept on the location and duration of turnout, management and veterinary‐attended episodes of disease or injury from birth until leaving the farm or study exit for 129 Thoroughbred foals on six stud farms. Dams' signalments, reproductive and gestational health records were collated concurrently. Available race performance records to the end of the fourth year of life were collected from industry databases. Mixed effects logistic and linear regression modelling, including farm, mare and stallion as random effects, were used to investigate associations between gestational and early‐life exposures and race performance outcomes.

**Results:**

Overall, 76% (98/129, 95% CI: 68–82) of horses raced, making a median of 7 starts (IQR 4–11, range 1–23) and earning a median of £6898 (IQR 1712–17,987; range 0–197,601) in prizemoney. Increasing average daily turnout time (hours) and turnout area (acres) in the first 6 months of life were associated with increased odds of racing and increased total prizemoney earned, respectively (OR 1.31, 95% CI: 1.09–1.58 *p* = 0.004 and *β*‐coefficient *=* 0.32 (lnGBP), 95% CI: 0.03–0.61, *p* = 0.03). Age at weaning (days) was associated with increased odds of racing and an increased total number of runs (OR 1.03, 95% CI: 1.01–1.05 and *β*‐coefficient = 0.09, 95% CI: 0.04–0.14, *p* < 0.05, respectively).

**Main Limitations:**

Absence of training‐related data; low study power for some exposure variables.

**Conclusions:**

More extensive early‐life turn out practices and later weaning enhanced later‐life race performance outcomes. This could be due to increased opportunity for positive musculoskeletal tissue adaptation and optimal growth and development rates during a critical window of developmental plasticity.

## Zusammenfassung

**Hintergrund:**

Expositionen während der Trächtigkeit und frühen Lebensphasen können die Entwicklung während des Wachstums und die zukünftige sportliche Leistungsfähigkeit beeifnflussen.

**Ziele:**

Untersuchung von Zusammenhängen zwischen pränatalen und früher Expositionen bei Vollblütern und (i) der Wahrscheinlichkeit zu starten, (ii) der Gesamtanzahl an Rennen sowie (iii) dem insgesamt erzielten Preisgeld bis zum Ende des dritten Lebensjahres.

Studiendesign: Prospektive Kohortenstudie.

**Methoden:**

Für 129 Vollblutfohlen auf sechs Gestüten wurden tägliche Aufzeichnungen zur Auslaufdauer und ‐fläche, Haltung sowie tierärztlich betreuten Erkrankungs‐ und Verletzungsereignissen von der Geburt bis zum Verlassen des Gestüts bzw. Studienaustritt geführt. Parallel wurden Angaben zur Abstammung sowie zur reproduktiven und trächtigkeitsbezogenen Gesundheit der Mütter erfasst. Leistungsdaten bis zum Ende des vierten Lebensjahres wurden aus brancheneigenen Datenbanken gesammelt. Zur Untersuchung der Zusammenhänge zwischen Expositionen und Leistungsparametern wurden gemischte logistische und lineare Regressionsmodelle mit Farm, Stute und Hengst als Zufallseffekte verwendet.

**Resultate:**

Insgesamt starteten 76 % der Pferde (98/129, 95 %‐KI: 68–82), mit einer Mediananzahl von 7 Rennen (IQR 4–11, Spanne 1–23) und einem Medianpreisgeld von £6.898 (IQR 1.712–17.987, Spanne 0–197.601). Eine längere durchschnittliche tägliche Auslaufzeit (in Stunden) und größere Auslauffläche (in Acres) in den ersten 6 Lebensmonaten waren mit einer erhöhten Wahrscheinlichkeit zu starten bzw. mit höherem erzieltem Preisgeld assoziiert (OR 1,31, 95 %‐KI: 1,09–1,58, P=0,004 und β‐Koeffizient=0,32 (lnGBP), 95 %‐KI: 0,03–0,61, P=0,03). Ein höheres Absetzalter (in Tagen) war mit einer erhöhten Startwahrscheinlichkeit sowie einer höheren Anzahl an Rennen verbunden (OR 1,03, 95 %‐KI: 1,01–1,05 und β‐Koeffizient=0,09, 95 %‐KI: 0,04–0,14, jeweils P<0,05).

## 1 INTRODUCTION

The concept of developmental plasticity, the ability of the adult phenotype to be altered via epigenetic mechanisms in response to exposures during gestation and early life, is well established in both human^1^ and equine medicine.^2^ Such alterations have the potential to confer long‐term effects, not only altering susceptibility to disease and injury in later‐life,^1^, ^3^ but potentially also influencing athletic performance.^2^

In studies of pregnant mares, maternal overnutrition and obesity,^4^ and the feeding of concentrate feed compared with forage‐only diets^5^ were associated with alterations in glucose metabolism in offspring up to 18 months of age^4^ and an increased risk of radiographic osteochondrosis lesions.^5^ Similarly, in utero growth restriction^6^ and shorter gestation length^7^ resulted in aberrations in offspring metabolic function^6^ and increased risk of developmental orthopaedic disease,^6^, ^7^ which may in turn impact athletic performance in horses.^8^, ^9^, ^10^ Advancing maternal age may restrict in utero growth and development via age‐related changes to uterine artery blood flow resistance and placental perfusion,^11^, ^12^ which may be why progeny from older mares have been associated with reduced race performance outcomes such as speed, Timeform rating and likelihood of winning stakes races, when compared with progeny from younger dams.^13^, ^14^, ^15^

In studies of foals during early life, moderate amounts of conditioning exercise over and above turn out resulted in increased bone size and strength and advanced the development of cartilage and tendon tissues.^16^, ^17^ In addition, more extensive turn out practices prior to weaning have been associated with a reduced risk of post‐weaning musculoskeletal disease and injury.^18^ Individuals receiving additional conditioning exercise were also able to complete, on average, more training sessions and more competitive events as 2‐year‐olds.^19^ In contrast, box rest, repetitive forced high intensity activity and aberration to turn out routines during early life had deleterious effects on musculoskeletal tissues^20^, ^21^, ^22^, ^23^ and increased the risk of developmental orthopaedic disease and musculoskeletal injury,^5^, ^18^ which have been associated with increased days lost from training^24^ and reduced race performance outcomes in Thoroughbreds.^10^, ^25^, ^26^

The aim of this study was, therefore, to investigate associations between gestational and early‐life exposures and race performance outcomes in flat‐race bred Thoroughbreds, specifically the (i) likelihood of racing, (ii) total number of runs and (iii) total prizemoney earned by the end of the fourth year of life (3‐year‐old‐year). We hypothesised that older dams, the use of restrictive turn out practices and episodes of musculoskeletal disease during early life would be associated with reduced race performance outcomes. Findings can be used to directly inform management strategies aimed at improving performance and reducing wastage in this population. The study reported here forms part of a large body of work, with further description of broodmare health and management,^27^ early‐life disease and injury,^28^ and associations between gestational and early‐life exposures and early‐life health outcomes^7^, ^18^ in this population published elsewhere.

## 2 MATERIALS AND METHODS

### 2.1 Study design and study period

Details of study recruitment are described in detail elsewhere.^28^ Briefly, a prospective cohort study was set up using a convenience sample of Thoroughbred studs across the United Kingdom and Ireland. All foals born on the recruited studs in 2019 entered the study and were under observation from birth until leaving the stud farm to enter training/pre‐training for racing at around 18–20 months of age or study exit. To increase the sample size, some studs also agreed to enrol all foals born in 2020; these foals were followed until 31 December 2020. Longer‐term follow‐up, which included collection of all available training, race performance, import and export, mortality and whereabouts data, was undertaken to the end of the fourth year of life (end of 3‐year‐old‐year), that is, 31 December 2022 for those born in 2019 and 31 December 2023 for those born in 2020.

### 2.2 Sample size calculation

Calculations were performed in Stata (Release 16, StataCorp LP). Using a regression approach to investigate associations between gestational and early‐life exposures and the odds of racing with 80% power and 5% type 1 error, it was estimated that data on 112 individuals (56 in each group; exposed and unexposed) would be required, assuming a 30% probability of exposure in the control group and an odds ratio of 3, increasing to 116 (58 in each group) with an exposure probability of 50% and to 274 (137 in each group) with an odds ratio of 2.

### 2.3 Data collection

#### 2.3.1 Gestational and early‐life exposures

Details of the methods of gestational and early‐life data collection are described in detail elsewhere.^7^, ^18^, ^27^, ^28^ In summary, between 1 January 2019 and 31 December 2020, farms were provided with customised daily recording booklets, in which they made coded entries identifying any days that foals received veterinary intervention for any reason, any medicine, or any management intervention (for example weaning). A subset of farms additionally recorded the location and duration of any turn‐out foals received. The area (in acres) of all turn‐out locations and further details of management interventions and episodes of veterinary‐attended injury or disease, along with dams' signalment, breeding history, reproductive management during the breeding season(s) and veterinary‐attended episodes of illness or injury and medication usage during gestation (date of last service to date of birth of the foal) were collated from farm and veterinary records. Details of the sire, last service date, dams' age, number of previous live foals and status at the start of the breeding season were verified using Weatherbys' Return of Mares.^29^

#### 2.3.2 Racing outcomes

Data to determine whether horses entered training with a registered trainer and raced at least once, anywhere in the world, before the end of the fourth year of life were collected from several data sources. Firstly, intermittent email follow‐up was undertaken with participating stud farms about the fate and whereabouts of individuals that remained under their ownership. Secondly, all available Weatherbys General Stud Book and British Horseracing Authority (BHA) data for individuals from the birth cohort between 1 January 2019 and 31 December 2023 were retrieved via a non‐disclosure agreement. Weatherbys' data included date of birth, dam, sire, sex, date of foal registration, naming and given name, and, where applicable, date of death, last available date of export and import along with the destination and whether this was permanent or temporary, and details of any appearances made at public auction. British Horseracing Authority data included date of birth, dam, sire, sex, name, details of registration with trainers licenced in Great Britain (GB) including date of registration and the name of the trainer, details of all race starts made in GB or abroad in races against GB horses including the date, racecourse, race type, placing and prizemoney won (in Great Britain Pounds (GBP)). Additionally, all individuals and their respective dams were manually searched for in publicly available data sources (listed in Data S1), from which any additional outcome data, such as race performances in foreign jurisdictions, occurring within the study period were collected. Race performance data (to calculate total number of starts and total prizemoney to the end of the fourth year of life) were collated solely from BHA databases for all horses that were recorded as having raced at least once. All data were entered into a custom designed Microsoft™ Access database.

### 2.4 Data processing

Data were imported into Stata (Release 16, StataCorp LP). To identify the study population in which to evaluate associations between gestational and early‐life exposures and performance outcomes, the cohort of yearlings that were alive at the end of the second year of life and eligible to enter training and race was established. Any individuals reported to have died on or before the end of the second year of life were excluded from the study dataset. The study population was further restricted to include only those foals for which early‐life turn out data were available, given our primary interest in evaluating early‐life turnout practices in relation to later‐life performance outcomes.

#### 2.4.1 Gestational and early‐life exposures

Processing of gestational exposure data are reported in detail elsewhere.^7^, ^27^ For the purposes of these analyses, mares age (years) at breeding was calculated as date of last service minus date of birth. Status at the start of the breeding season was categorised as foaling (mare produced a live foal from the previous breeding season), maiden (mare had never produced a live foal) or barren (mare had failed to produce a live foal in the previous season but had previously produced one or more live foals). For all non‐maiden mares, the number of previous live foals produced by the mare prior to each breeding season was calculated from the mares' return outcomes reported in Weatherbys' Return of Mares.^29^ Gestation length was calculated as foaling date minus last service date. Episodes of mare illness and injury and medications prescribed during gestation were categorised, using diagnoses and clinical descriptions recorded in veterinary records, which are described elsewhere.^27^ For the purposes of these analyses, variables were created to identify mares that had suffered at least one episode of disease or injury during gestation that required veterinary intervention and mares that had received at least one medication during gestation.

Processing of early‐life disease and injury event data and turn out data are also described in detail elsewhere.^7^, ^18^, ^28^ For the purposes of these analyses, foals' month and year of birth were derived from the foals' date of birth. Age at weaning was calculated as date of weaning minus date of birth. Variables were created to indicate whether individuals attended foal and/or yearling sales and identify foals that had received veterinary intervention at least once between birth and the end of the second year of life for developmental orthopaedic disease (DOD), musculoskeletal trauma, conditions affecting the foot, miscellaneous musculoskeletal conditions, colic, pneumonia and/or enteritis/colitis. Case definitions for these categories are described elsewhere.^28^ Utilising cumulative totals of the daily turn out time (hours) and turn out area (acres), an average daily turn out time and area exposure variable for each 30‐, 90‐ and 180‐day period of foal age during the first 12 months of life were created. Turn out time and area were recorded as 0 on days on which foals did not receive any turn out (i.e., box rest), and days when foals were away from the farm for any reason (for example for the mare to be covered) and therefore not under observation were not included. An exposure variable was also created that recorded the age at which foals first received 24/7 turn out.

#### 2.4.2 Race performance outcomes

Outcome variables were created to indicate whether individuals had been registered with a trainer or raced at least once, anywhere in the world, before the end of the fourth year of life (i.e., end of the 3‐year‐old year). For the subset of horses that had at least one race start recorded in BHA databases, outcome variables were created to indicate the total number of runs and the total prizemoney won during the study period.

### 2.5 Data analysis

Continuous data were described using mean, standard deviation (SD) and range if normally distributed and median, inter‐quartile range (IQR) and range if non‐normally distributed. Proportions and 95% confidence intervals (CI) were calculated for categorical data.

To investigate associations between gestational and early‐life exposures and the odds of racing, a mixed effects multivariable logistic regression model was constructed, in which nine gestational, 10 early‐life health and management and 37 early‐life turn out exposures were evaluated as fixed effects, and farm, mare and stallion were evaluated as random effects. To investigate associations between gestational and early‐life exposures and the total number of races and total prizemoney earned, mixed effects multivariable linear regression models were constructed, evaluating the same fixed and random effects. Due to non‐normality and heteroscedasticity in the error term (residuals) total prizemoney earned was log transformed for modelling purposes.

The shape of the association between continuous exposure variables and the outcome of interest was explored using a likelihood ratio test (LRT) for departure from linear trend, with variables modelled as categorical (quartiles) if LRT *p* < 0.05. For turn out variables under such circumstances, categories were created based on data distribution and knowledge of farm turn out practices as per methods previously described elsewhere.^18^ Random effects (potential cluster variables; farm, mare and stallion) were retained if a LRT *p*‐value comparing a model with the random effect to a simple logistic or linear model was <0.05; otherwise, simple logistic/linear models were used.

Variables with a univariable LRT *p* < 0.20 were taken forward to be considered for inclusion in a multivariable model. Multivariable models were built using a forward stepwise approach with inclusion based on a LRT *p* < 0.05. There was deemed to be evidence of confounding if odds ratio/coefficient estimates changed by >20% with inclusion of the second variable in the model, in which case the confounding variable was retained in the model. Two‐way interaction was evaluated between all variables retained in the multivariable model, and then between these variables and all other variables with a univariable *p* < 0.20. Interaction was deemed to be present, and the term retained, if the LRT comparing a model with the interaction term to a model without resulted in *p* < 0.05. In the case where turn out data describing different periods of foal age were eligible to be taken forward, separate models were built using either 30‐, 90‐ or 180‐day variables and the one with the lowest Akaike Information Criterion (AIC) was retained. The normality and homoscedasticity of the residuals were confirmed graphically using *Q*–*Q* plots and plots of residuals against the predicted values. Model specification was evaluated using a linktest.^30^

To further understand relationships between variables in the final models and the respective outcomes, results are presented as predicted margins^31^ the predicted mean probability of racing for logistic models and the predicted mean number of races and predicted mean total prizemoney for linear models. The potential bias of reverse transformation from lnGBP to GBP for the prizemoney margins estimates was accounted for by including a function of the variance of errors in all predictions.^32^

## 3 RESULTS

### 3.1 Description of study population and exposure variables

Gestational and early‐life turn out and management data were available for 129 yearlings (68 colts and 61 fillies) that were alive at the end of the second year of life and eligible to enter training. Yearlings were sired by 64 stallions and born from 121 mares on six stud farms across the United Kingdom (110 in 2019 and 19 in 2020). The distribution of the study population across gestational and early‐life health and management exposure variables is presented in Tables [Link], [Link]. Detailed descriptions of gestational and early‐life health and management exposure data are reported elsewhere.^7^, ^18^, ^27^, ^28^

### 3.2 Description of race performance outcomes

By the end of the fourth year of life, 95.3% (123/129; 95% CI: 90.2–97.8) of horses were registered with a trainer and 76.0% (98/129; 95% CI: 67.9–82.5) had raced at least once. British Horseracing Authority race performance data were available for 84 of the 129 study horses (44 colts and 40 fillies born from 79 mares on six stud farms across the United Kingdom, sired by 48 stallions). The median total number of runs by the end of the 3‐year‐old year for this subset was 7 (IQR 4–11; range 1–23) with a median total prizemoney of £6898 (IQR 1712–17,987; range 0–197,601). The distributions of race performance outcomes by exposure are presented in Tables [Link], [Link]. The 14 horses that had raced but for which no BHA performance data were available all trained and raced outside of GB.

### 3.3 Associations between gestational and early‐life exposures and race performance outcomes

#### 3.3.1 Racing at least once

Univariable results are presented in Table S1; the final multivariable model is presented in Table 1.

**TABLE 1:** Results of multivariable logistic regression analysis to investigate associations between gestational and early‐life exposures and the likelihood of racing at least once by the end of the fourth year of life in a cohort of 129 flat‐bred Thoroughbreds born on six stud farms across the United Kingdom between 1 January 2019 and 31 December 2020.

Predictor	Odds ratio	95% confidence interval		Wald *p*	LRT *p*
Average daily turn out time in the first 6 months of life (hours)	1.31	1.09	1.58	0.004	0.004
Age at weaning (days)^a^	1.03	1.00	1.05	0.03	0.02

Abbreviation: LRT, likelihood ratio test.

^a^ Also a confounder of the relationship between average daily turnout time in the first 6 months of life and the odds of racing.

**FIGURE 1:**
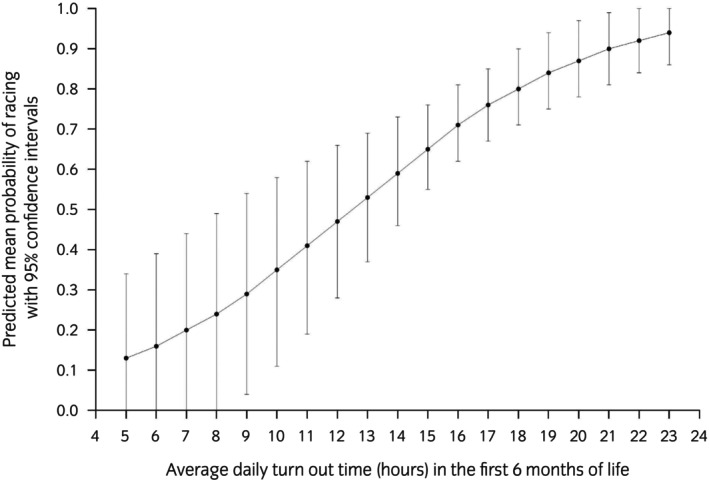
Predicted mean probability of racing at least once by the end of the fourth year of life over the observed distribution of average daily turn out time (hours) in the first 6 months of life for 129 flat race‐bred Thoroughbreds born on six stud farms across the United Kingdom in 2019 and 2020.

The average daily turn out time in the first 6 months of life and age at weaning were positively associated with the odds of racing at least once by the end of the fourth year of life. Figure 1 presents the predicted mean probability of racing at least once over the range of average daily turn out time during the first 6 months of life observed in the study population, after adjusting for the effect of age at weaning. Individuals who spent more time turned out during the first 6 months of life had a higher predicted probability of racing at least once by the end of their 3‐year‐old year. The predicted probability of racing was around 24% for foals that were turned out during the day and in at night for the whole 6‐month period (average daily turn out time of 8 h or less, Figure 1) and around 70% for those who spent the first months of life in at night and out during the day, with turn out times slowly increasing to being turned out 24/7 for the remaining months (average daily turn out time of around 16 h, Figure 1).

**FIGURE 2:**
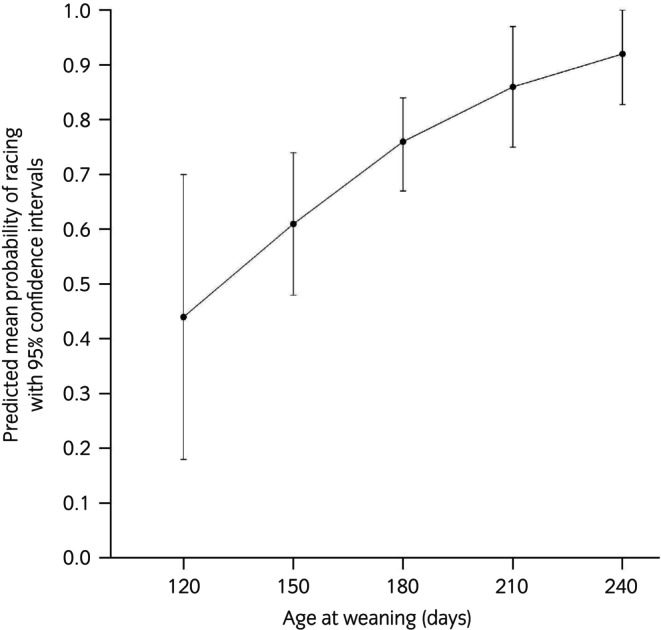
Predicted mean probability of racing at least once by the end of the fourth year of life over the observed distribution of age (days) at weaning for 129 flat race‐bred Thoroughbreds born on six stud farms across the United Kingdom in 2019 and 2020.

Figure 2 presents the predicted mean probability of racing at least once over the range of ages at weaning observed in the study population, after adjusting for the effect of the average daily turn out time in the first 6 months of life. Individuals weaned at an older age had a higher predicted probability of racing, which varied from just over 40% for individuals weaned at 4 months of age to almost 90% for those weaned at 8 months of age. Age at weaning was a confounder of the relationship between the average daily turn out time in the first 6 months of life and the odds of racing at least once. No interaction was detected. Farm, mare and stallion were not associated with the odds of racing at least once. A link test supported correct model specification (*p* = 0.74) and graphs demonstrating the normality and homoscedasticity of the residuals are provided in Figure S1.

#### 3.3.2 Total number of races

Univariable results are presented in Table S2; the final multivariable model is presented in Table 2.

**TABLE 2:** Results of multivariable linear regression analysis to investigate associations between gestational and early‐life exposures and the total number of race starts made by the end of the fourth year of life in a cohort of 84 flat‐bred Thoroughbreds born on six stud farms across the United Kingdom in 2019 and 2020.

Predictor	Coefficient	95% confidence interval		Wald *p*	LRT *p*
Age at weaning (days)	0.09	0.04	0.14	0.001	0.001
Month of birth^a^					
January	−5.28	−10.43	−0.12	0.04	0.16
February	0.31	−2.71	3.33	0.84	
March	*Ref*				
April	1.05	−2.46	4.57	0.55	
May	2.57	−2.66	7.80	0.33	

Abbreviation: LRT, likelihood ratio test.

^a^ Confounder of the relationship between age at weaning and the total number of race starts made by the end of the fourth year of life.

**FIGURE 3:**
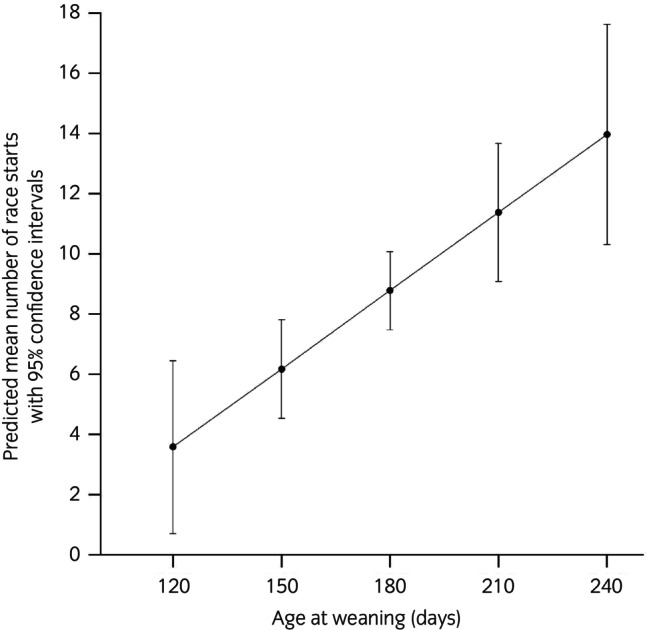
Predicted mean number of race starts (after adjusting for the effect of month of birth) made by the end of the fourth year of life over the observed distribution of age at weaning for a population of 84 flat‐race bred Thoroughbreds born on six stud farms across the United Kingdom in 2019 and 2020.

Age at weaning was positively associated with the total number of races by the end of the fourth year of life. Month of birth was a confounder of the relationship between age at weaning and number of races. Figure 3 presents the predicted mean number of races by the end of the fourth year of life from the final model over the distribution of age at weaning observed in the study population, after adjusting for the effect of month of birth. Increasing age at weaning was associated with an increased predicted total number of races. Individuals weaned at 4 months of age were predicted to compete in a mean of ~4 races, compared with a mean of around 6, 9, 11 and 13 races for individuals weaned at 5, 6, 7 and 8 months of age, respectively. No interaction was detected. Farm, mare and stallion were not associated with the total number of races by the end of the fourth year of life. A link test supported correct model specification (*p* = 0.41) and graphs demonstrating the normality and homoscedasticity of the residuals are provided in Figure S1.

#### 3.3.3 Total prizemoney

Univariable results are presented in Table S3 and the final multivariable model is provided in Table 3.

**TABLE 3:** Results of multivariable linear regression analysis to investigate associations between gestational and early‐life exposures and the natural log of the total prizemoney earned by the end of the fourth year of life in a cohort of 84 flat‐bred Thoroughbreds born on six stud farms across the United Kingdom in 2019 and 2020.

Predictor		Coefficient	95% confidence interval		Wald *p*	LRT *p*
Developmental orthopaedic disease	No	*Ref*				0.01
	Yes	−0.77	−1.89	0.34	0.17	
Average daily turn out area in the first 6 months of life (acres)^a^		0.32	0.03	0.61	0.03	0.02

Abbreviation: LRT, likelihood ratio test.

^a^ Also a confounder of the relationship between DOD and the total prizemoney won by the end of the third year of life.

**FIGURE 4:**
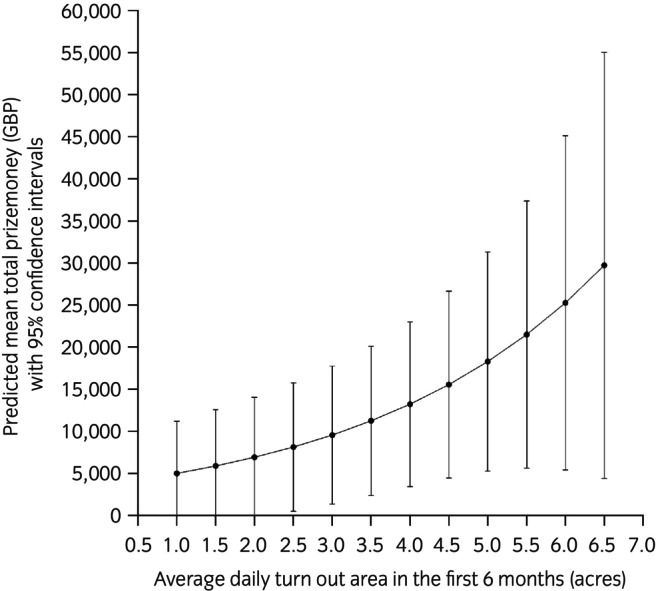
Predicted mean total prizemoney (Great Britain Pounds [GBP]) earned by the end of the fourth year of life over the observed distribution of the average daily turn out area (acres) in the first 6 months of life (after accounting for any effect of early‐life developmental orthopaedic disease) for a population of 84 flat‐race bred Thoroughbreds born on six stud farms across the United Kingdom in 2019 and 2020.

The average daily turn out area (acres) in the first 6 months of life and whether the horse had required veterinary intervention for DOD in the first 2 years of life were associated with the natural log of total prizemoney earned (lnGBP) by the end of the fourth year of life. The average daily turn out area in the first 6 months of life was also a confounder of the relationship between DOD and the natural log of the total prizemoney earned by the end of the second year of life and, once the effects of early‐life turn out area were accounted for in the model, the effect of DOD was not significant (Wald *p* = 0.17, Table 3). Figure 4 presents the predicted mean prizemoney earned over the range of average daily turn out areas in the first 6 months of life observed in the cohort, after adjusting for any effect of DOD. As the average turn out area increased, so did the predicted prizemoney (GBP). Individuals that had restricted turn out during early‐life (average of 2 acres or less, Figure 4) were estimated to earn less than £7000 in prizemoney, compared with those exposed to more extensive turn out (average daily turn out area of 4 acres or more, Figure 4), who were estimated to earn over £13,000 in prizemoney. After accounting for the effects of early‐life turn out area, differences in predicted mean prizemoney earned between individuals with and without DOD were not significant (*p* > 0.05 in pairwise comparisons of predicted margins; predicted mean difference DOD vs. non‐DOD £12,369; 95% CI: £29,532–£4784). No interaction was detected. Farm, mare and stallion were not associated with the total prizemoney earned by the end of the fourth year of life when evaluated as random effects. A link test supported correct model specification (*p* = 0.51) and graphs demonstrating the normality and homoscedasticity of the residuals are provided in Figure S1.

## 4 DISCUSSION

This study is the first to utilise prospective, observational birth cohort data to investigate associations between gestational and early‐life exposures and later‐life performance outcomes in UK Thoroughbreds. It provides novel findings that can be directly applied at the stud farm level to enhance future performance. Findings suggest that the use of more extensive turn‐out practices during the first 6 months of life and later weaning may be of benefit in terms of attainment of career milestones, increased productivity (number of races) and financial returns in this population.

The ability of early‐life activity and exercise to modulate musculoskeletal tissue development has been previously demonstrated in small, experimental studies.^17^, ^19^, ^20^ These suggest that there is likely to be an optimal workload, dependent on age (stage of development), at which concurrent tissue adaptations may have the potential to confer positive consequences in terms of future disease and injury risk.^16^, ^17^, ^20^, ^21^, ^22^, ^23^ Previous work from the present cohort provided further evidence to this effect by demonstrating associations between early‐life turn out practices (in the first 6 months of life) and the risk of musculoskeletal disease and injury between 12 and 18 months of age.^18^ However, prior to the present study, any effects of these exposures on later‐life health and performance outcomes have not been evaluated outside of experimental studies.^19^ Given that Thoroughbreds' only opportunities to realise financial returns to their production costs are either by sale or through revenue generated during their racing career, findings from studies evaluating the achievement of this milestone alongside opportunities to earn prizemoney could aid productivity and profitability, which are of ever‐increasing importance in the current financial climate facing UK Thoroughbred breeders.^33^ The benefit of observational studies such as the present work is that the evidence base they generate can be directly applied at horse‐ and farm level to inform stud farm management strategies.

In the present study, increasing turn out time and area in the first 6 months of life were associated with an increased likelihood of racing and an increased total prizemoney earned by the end of the fourth year of life, respectively. The first 6 months of life, prior to weaning, are the period of most rapid post‐natal growth in Thoroughbreds, correspondent to the period of rapid infant growth in humans.^34^ During this period, there is rapid longitudinal growth of the distal limb, gain in bodyweight, and development of muscle mass, with individuals reaching up to 43% of adult bodyweight and 83% of adult height.^34^ This phase is also an important period of developmental plasticity, where tissues' sensitivity to environmental exposures is likely to be high.^1^, ^35^ Studies utilising GPS technology to record the activities and workload of both feral^36^, ^37^ and domesticated foals kept extensively at pasture^38^ during this period have demonstrated that although total workload (distance × speed) decreases as the amount of time spent grazing increases, the frequency of high‐intensity activities such as cantering, galloping, rearing and bucking increases. It is postulated that these high‐intensity locomotor activities play an important role in musculoskeletal development by stimulating positive adaptation of tissues for the given stage of development of the foal.^38^, ^39^ It is well established that factors such as paddock size,^40^ turn out time and turn out routine influence activity levels and behaviours exhibited at pasture.^38^, ^40^, ^41^, ^42^, ^43^, ^44^, ^45^, ^46^, ^47^ Therefore, we hypothesise that individuals in the present study who spent more time at pasture in larger paddocks during this period of rapid growth and developmental plasticity had a greater opportunity to achieve the necessary workloads and activity intensity for positive musculoskeletal tissue adaptation, which in turn led to enhanced resistance to injury and disease and athletic performance in later life.

Early‐life exercise and activity may not only confer positive effects on musculoskeletal tissues. In human and rodent studies, early‐life activity and exercise have been associated with improved activity motivation,^48^, ^49^ improved metabolic function and reduced obesity,^49^, ^50^, ^51^ and improved anti‐inflammatory immunity^49^ in adulthood. Such traits are likely to be of benefit, not only in terms of health but also athletic performance. Childhood motor and cardiorespiratory fitness have also been shown to enhance cognition and lower perceived stress during adolecence,^52^ factors which could be beneficial to young Thoroughbreds in terms of trainability, given that unsuitable temperament and behaviour have been cited as reasons for failure to train and race.^53^ The early‐life social environment also plays an important role in the development of coping behaviours,^54^ and foals spending more time turned out in larger areas (turn out area was highly correlated to turn out group size in this population^18^) during early life will have had more access to social interactions with individuals other than their dams. Alongside encouraging necessary juvenile play behaviours and high intensity locomotor activities,^38^, ^39^ this could also play an important role in their cognitive development and later‐life resilience^54^ in turn, perhaps enhancing their performance.

Age at weaning was positively associated with both the likelihood of racing and the total number of race starts by the end of the fourth year of life. On all farms in the present study, weaning was undertaken by abrupt removal of the dam, with foals frequently being stabled with reduced turnout during this period. As such, age at weaning was also associated (confounder) with the average daily turnout time during the first 6 months of life and, therefore, some of the effect of early weaning on the likelihood of racing in this population could have been as a result of the associated reductions in average daily turnout time in the first 6 months of life.

Abrupt weaning, as was undertaken in the present study, is recognised to cause a significant reduction in growth rate (average daily weight gain),^55^ which may in turn slow the early‐life phase of rapid growth and development.^34^ Therefore, particularly when it is undertaken early, weaning could potentially reduce the window of developmental plasticity and opportunity for positive tissue adaptation. In studies comparing bone mineral content (BMC)^56^ and canon circumference growth^55^ of early‐ (~4 months of age) and later‐weaned (~6 months of age) foals, early‐weaned foals exhibited a weaning‐associated reduction in BMC gain and canon circumference growth in the 4–6 weeks post weaning, which was not observed in the later‐weaned foals. This supports the hypothesis that weaning during this critical period of tissue plasticity could reduce the potential for positive adaptation. Further work is clearly required to test this hypothesis; however, the fact that early weaning was also associated with a reduced number of races, a potential proxy of later‐life soundness and career longevity, provides some evidence to support this theory.

Weaning is recognised as being one of the most stressful events in the life of a horse.^57^, ^58^ In both humans and rodents, early‐life stress induced by maternal separation (rodents) or neglect (humans) alters hypothalamic–pituitary–adrenal axis activity and leads to altered glucose metabolism, insulin resistance and obesity in later life,^59^, ^60^ conditions which are recognised to negatively affect athletic performance in horses.^8^ Evidence from observational studies of naturalistic equine populations suggests that when foals were allowed to wean voluntarily, which occurred on average at around 9–10 months of age, any weaning‐associated stress response appeared to be alleviated.^61^ Given present findings, studies to further elucidate the effects of weaning and the impact of management around this time in Thoroughbreds are warranted and could help further inform strategies to enhance later life health and performance in this population.

It was hypothesised that early‐life disease, particularly of the musculoskeletal system, would itself have a negative impact on later‐life performance outcomes in the present population. In support of this, requiring veterinary intervention for a developmental orthopaedic condition before the end of the second year of life was associated with the total prizemoney earned by the end of the fourth year of life. However, once early‐life turn out strategies had been accounted for (the average daily turn out area in the first 6‐months of life, was a confounder of this relationship), the effect of DOD was no longer significant. In the source population for this study, half of all individuals diagnosed with DOD required veterinary intervention in the first 6‐months of life,^28^ with the majority (74%) of cases being described as flexural deformities.^28^ Box rest or restricted turn out are commonly prescribed in the management of these conditions^62^ and for acquired deformities, where energy intake is advised to be reduced in rapidly growing foals,^63^ farms may elect to undertake weaning. As such, it may be that the greatest risk such conditions pose to later‐life race performance is via restrictions in early‐life turn out and early weaning undertaken as part of their management. It may therefore be prudent, when selecting treatment and management strategies for these conditions during early‐life, to carefully consider the balance between restriction of exercise and growth and the provision of adequate opportunity for tissue adaptation during this critical window of developmental plasticity.

Conventionally, race performance outcomes in Thoroughbreds are often attributed to genetic factors, particularly pedigree, which has led to top‐pedigree individuals consistently achieving a premium at sales and breeders making large investments in covering fees to utilise the most desirable sires.^64^, ^65^ It is, therefore, interesting that no significant variation in race‐performance outcomes was observed when either stallion or mare was evaluated as random effects in the present study, once early‐life exposure factors had been accounted for. In humans, it is recognised that to maximise athletic potential a positive interaction between gene and environment is required.^66^ However, it is also understood that gene and environment can often be correlated, in that genetically gifted individuals may be identified as children and consequently provided with an optimised environment that further aids them in achieving their maximum potential.^66^ This may well be similar in Thoroughbreds, where greater investments in early‐life management might be made in higher value individuals or those perceived to have greater genetic potential.

The main limitations of this study are the relatively small sample size and the lack of health and management data during training. Particularly for race‐performance outcomes, some analyses may have lacked power; several factors with the potential to influence race performance outcomes were not accounted for in present models. Additionally, the use of BHA race performance data is likely to underestimate the number of overseas race starts, as it does not include data from horses trained outside of GB unless they ran against at least 1 GB trained horse.

In conclusion, the first 6 months of life is a critical period of developmental plasticity, in which it is essential to ensure that turn out and weaning practices provide sufficient opportunity for positive tissue adaptation and development. Further investigation into weaning methods and strategies to reduce the need for restriction of exercise and early weaning in the management of developmental orthopaedic conditions in this population is warranted in the context of improving later‐life performance outcomes.

## FUNDING INFORMATION

Funding for this study was provided by the Horserace Betting Levy Board (EPDF 2022‐9 and VET/PRJ/791), the Racing Foundation and the Royal Veterinary College's Mellon Fund for Equine Research.

## CONFLICT OF INTEREST STATEMENT

The authors have declared no competing interests.

## AUTHOR CONTRIBUTIONS

**Rebecca Mouncey:** Conceptualization; funding acquisition; project administration; formal analysis; software; data curation; writing – review and editing; visualization; validation; methodology; investigation; writing – original draft. **Amanda M. de Mestre:** Conceptualization; funding acquisition; project administration; supervision; resources; methodology; writing – review and editing. **Juan Carlos Arango‐Sabogal:** Conceptualization; formal analysis; resources; writing – review and editing. **Kristien L. Verheyen:** Conceptualization; funding acquisition; project administration; writing – review and editing; methodology; resources; supervision.

## DATA INTEGRITY STATEMENT

Rebecca Mouncey had full access to all the data in the study and takes responsibility for the integrity of the data and the accuracy of the data analysis.

## ETHICAL ANIMAL RESEARCH

Ethical approval was granted by the Royal Veterinary College's Clinical Research Ethical Review Board (URN: 2024 2251‐2).

## INFORMED CONSENT

Informed consent was obtained for inclusion of animals in this study. Weatherbys General Stud Book and British Horseracing Authority race performance data were collected under a non‐disclosure agreement.

## Supporting information


**Data S1.** List of publicly available data sources from which follow‐up data were collected.

**Figure S1.** Graphs demonstrating the normality and homoscedasticity of the residuals from the final multivariable models investigating associations between gestational and early‐life exposures and race performance outcomes in a cohort of 129 flat‐bred Thoroughbreds born on six stud farms across the United Kingdom between 1 January 2019 and 31 December 2020.

**Table S1.** The distribution of the probability of racing at least once by the end of the fourth year of life by exposure and results of univariable logistic regression analysis to investigate associations between gestational and early‐life exposures and the likelihood of racing at least once by the end of the fourth year of life, in a cohort of 129 flat‐bred Thoroughbreds born on six stud farms across the United Kingdom between 1 January 2019 and 31 December 2020.

**Table S2.** The distribution of the total number of race starts made by the end of the fourth year of life by exposure and results of univariable logistic regression analysis to investigate associations between gestational and early‐life exposures and the total number of race starts made by the end of the fourth year of life, in a cohort of 84 flat‐bred Thoroughbreds born on six stud farms across the United Kingdom between 1 January 2019 and 31 December 2020.

**Table S3.** The distribution of the total prizemoney won (GBP) by the end of the fourth year of life by exposure and results of univariable logistic regression analysis to investigate associations between gestational and early‐life exposures and the natural logarithm of the total prizemoney won (lnGBP) by the end of the fourth year of life, in a cohort of 84 flat‐bred Thoroughbreds born on six stud farms across the United Kingdom between 1 January 2019 and 31 December 2020.

## Data Availability

The data that support the findings of this study are available from Weatherbys General Stud Book and British Horseracing Authority. Restrictions apply to the availability of these data, which were used under licence for this study.
