# Supplementary material for: Born to run? Associations between gestational and early‐life exposures and later‐life performance outcomes in Thoroughbreds
Source: Equine Vet J. 2025 Aug 25;58(4):1071–81. doi: 10.1111/evj.70084 (PMC13244176; doi:10.1111/evj.70084)
Supplement: Supplementary file 1 — Data S1. List of publicly available data sources from which follow‐up data were collected. [file EVJ-58-1071-s002.pdf]

Data S1: List of publicly available data sources from which follow-up data were collected.

Data Source	Available at	Accessed
RacingPost Bloodstock	https://www.racingpost.com/bloodstock/	Jan-24
Racing and Sports	https://www.racingandsports.com.au/	Jan-24
Tattersalls	https://www.tattersalls.com/	Jan-24
Tattersalls Ireland	https://www.tattersalls.ie/	Jan-24
Goffs	https://www.goffs.com/	Jan-24
Arquana	https://www.arqana.com/	Jan-24
France Gallop	https://www.france-galop.com/en	Jan-24
German Studbook and Sales	https://bbag-sales.de/home~en	Jan-24
Japanese Studbook and Racing	https://www.studbook.jp/users/en/UserMenu.php	Jan-24
The Jockey Club of the Czech Republic	http://www.dostihyjc.cz/	Jan-24
The Turkish Jockey Club	https://www.tjk.org/	Jan-24
Polish Jockey Club	https://koniewyscigowe.pl/	Jan-24
