# Supplementary material for: Born to run? Associations between gestational and early‐life exposures and later‐life performance outcomes in Thoroughbreds
Source: Equine Vet J. 2025 Aug 25;58(4):1071–81. doi: 10.1111/evj.70084 (PMC13244176; doi:10.1111/evj.70084)

Equine Veterinary Journal BEVA

Figure S1: Graphs demonstrating the normality and homoscedasticity of the residuals from the final multivariable models investigating associations between gestational and early-life exposures and race performance outcomes in a cohort of 129 flat-bred Thoroughbreds born on six stud farms across the United Kingdom between 1 January 2019 and 31 December 2020.

(i) RACING AT LEAST ONCE MODEL

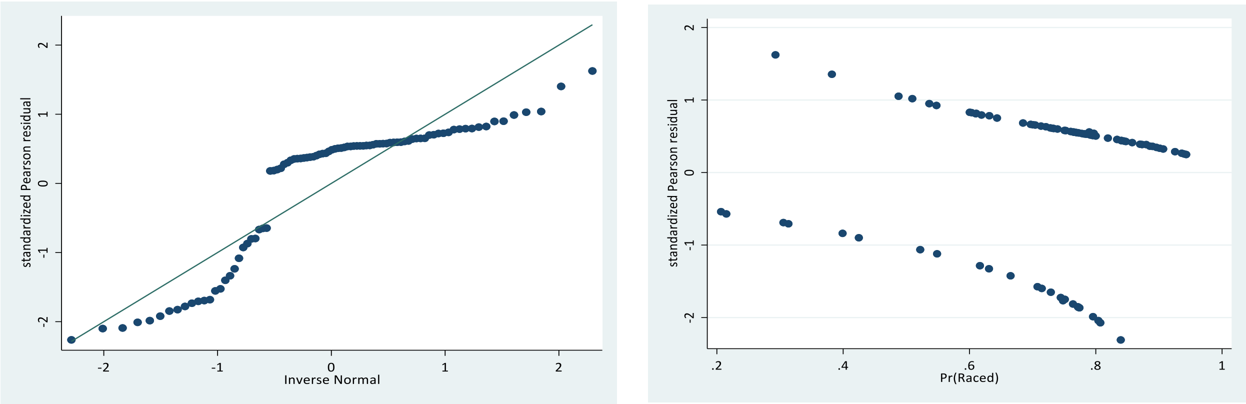

(ii) TOTAL NUMBER OF RUNS MODEL

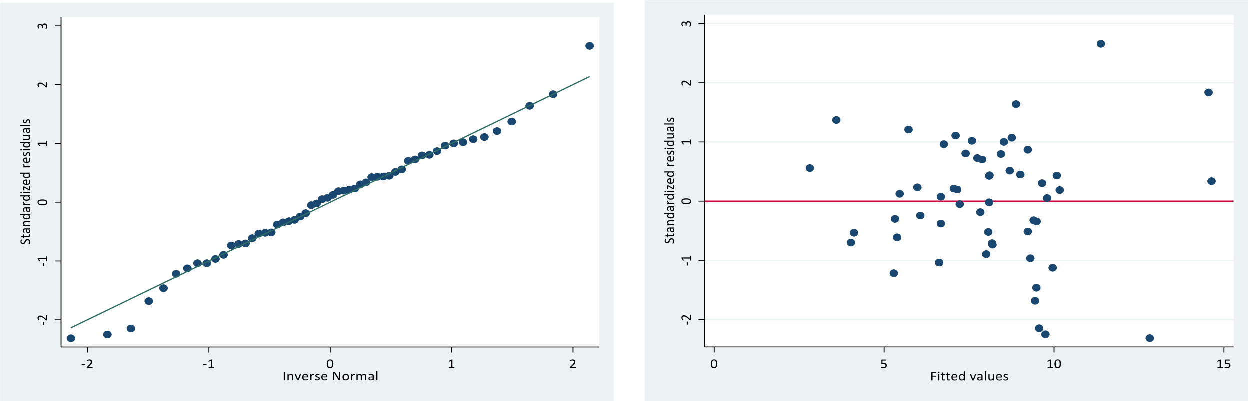

(iii) TOTAL PRIZEMONEY MODEL

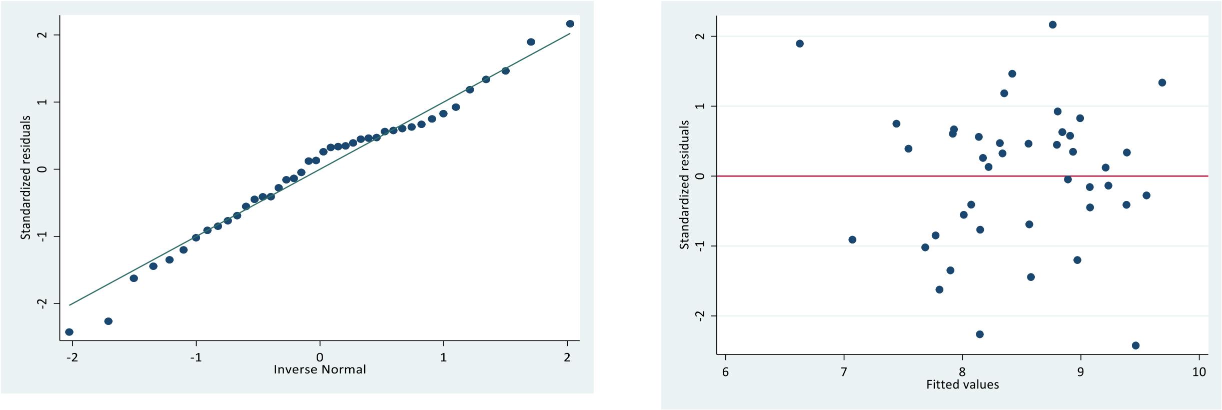

Supplement: Supplementary file 2 — Figure S1. Graphs demonstrating the normality and homoscedasticity of the residuals from the final multivariable models investigating associations between gestational and early‐life exposures and race performance outcomes in a cohort of 129 flat‐bred Thoroughbreds born on six stud farms across the United Kingdom between 1 January 2019 and 31 December 2020. [file EVJ-58-1071-s005.pdf]
