# Supplementary material for: Born to run? Associations between gestational and early‐life exposures and later‐life performance outcomes in Thoroughbreds
Source: Equine Vet J. 2025 Aug 25;58(4):1071–81. doi: 10.1111/evj.70084 (PMC13244176; doi:10.1111/evj.70084)
Supplement: Supplementary file 3 — Table S1. The distribution of the probability of racing at least once by the end of the fourth year of life by exposure and results of univariable logistic regression analysis to investigate associations between gestational and early‐life exposures and the likelihood of racing at least once by the end of the fourth year of life, in a cohort of 129 flat‐bred Thoroughbreds born on six stud farms across the United Kingdom between 1 January 2019 and 31 December 2020. [file EVJ-58-1071-s001.pdf]

Equine Veterinary Journal BEVA

Table S1: The distribution of the probability of racing at least once by the end of the fourth year of life by exposure and results of univariable logistic regression analysis to investigate associations between gestational and early-life exposures and the likelihood of racing at least once by the end of the fourth year of life, in a cohort of 129 flat-bred Thoroughbreds born on six stud farms across the United Kingdom between 1 January 2019 and 31 December 2020.

**RACING**									
	**DESCRIPTIVE STATISTICS**				**UNIVARIABLE**				
**FOAL ATTRIBUTES**	**not-raced**	**raced**	**total**	**% raced**	**OR**	**95%CI**		**Wald P**	**P**
SEX									
Colt	15	53	68	0.78	**ref**				0.56
Filly	16	45	61	0.74	0.78	0.34	1.71	0.56	
MONTH OF BIRTH									
January	2	7	9	0.79	0.70	0.12	4.01	0.69	0.51
February	11	24	35	0.69	0.43	0.15	1.23	0.12	
March	8	40	48	0.83	**ref**				
April	7	22	29	0.76	0.63	0.2	1.96	0.42	
May	3	5	8	0.63	0.33	0.07	1.68	0.18	
YEAR OF BIRTH									
2019	26	84	110	0.76	**ref**				0.89
2020	5	14	19	0.74	0.91	0.27	3.07	0.89	
GESTATION LENGTH									
	median	IQR	range						
	341	333 to 349	314 to 397						
314-337	14	33	47	0.7					
238-343	3	23	26	0.88					
344-351	7	22	29	0.76					
352-397	5	20	25	0.8					

per day					1.01	0.98	1.04	0.48	0.48
**EARLY-LIFE MANAGEMENT**									
AGE AT WEANING									
	median	IQR	range						
	170	155 to 179	112 to 250						
112-155	8	17	25	0.68					
156-170	9	15	24	0.62					
171-179	5	18	23	0.78					
180-250	6	17	23	0.73					
per day					1.02	0.99	1.04	0.19	**0.19**
SOLD AS A FOAL									
no	26	90	116	0.78	**ref**				0.22
yes	5	8	13	0.61	0.48	0.14	1.59	0.22	
SOLD AS A YEARLING									
no	20	50	70	0.71	**ref**				**0.19**
yes	31	48	59	0.81	1.71	0.76	4.02	0.19	
AGE FIRST TURNED OUT 24/7									
	median	IQR	range						
	80	65 to 98	32 to 174						
32 to 65	9	16	25	0.64					
66 to 80	6	21	27	0.78					
81 to 98	4	19	23	0.83					
99 to 174	10	15	25	0.60					

per day					0.99	0.98	1.01	0.45	0.45
AVERAGE DAILY TURN OUT TIME MONTH 1 (hours)									
	median	IQR	range						
	3	2 to 5	0 to 7						
0 to 2	13	31	44	0.70					
3 to 5	9	35	34	0.73					
6 to 7	8	36	44	0.81					
per hour					1.02	0.95	1.53	0.12	**0.12**
AVERAGE DAILY TURN OUT TIME MONTH 2									
	median	IQR	range						
	6	5 to 7	1 to 23						
1 to 5	10	20	30	0.67					
6 to 7	5	19	24	0.79					
8 to 23	11	27	38	0.71					
per hour					0.99	0.89	1.1	0.82	0.82
AVERAGE DAILY TURN OUT TIME MONTH 3									
	median	IQR	range						
	17	8 to 22	2 to 24						**0.13**
1 to 8	12	32	33	0.63	0.96	0.35	2.67	0.94	
9 to 23	4	24	28	0.86	3.3	0.91	11.97	0.07	
24	11	20	31	0.64	**ref**				

AVERAGE DAILY TURN OUT TIME MONTH 4									
	median	IQR	range						
	24	21 to 24	5 to 24						
1 to 8	5	5	10	0.50					
9 to 23	10	37	47	0.79					
24	13	26	39	0.67					
per hour					1.06	0.98	1.14	0.16	**0.16**
AVERAGE DAILY TURN OUT TIME MONTH 5									
	median	IQR	range						
	24	24 to 24	3 to 24						
1 to 8	3	0	3	0.00					
9 to 23	10	32	42	0.76					
24	15	35	50	0.7					
per hour					1.15	0.99	1.33	0.06	**0.06**
AVERAGE DAILY TURN OUT TIME MONTH 6									
	median	IQR	range						
	24	23 to 24	3 to 24						
1 to 8	2	1	3	0.33					
9 to 23	11	28	39	0.72					
24	15	38	53	0.72					
per hour					1.15	0.97	1.36	0.11	**0.11**
AVERAGE DAILY TURN OUT TIME MONTH 7									

	median	IQR	range						
	24	23 to 24	3 to 24						
1 to 8	2	0	2	0.00					
9 to 23	10	28	38	0.74					
24	14	36	50	0.72					
per hour					1.19	0.98	1.43	0.08	**0.08**
AVERAGE DAILY TURN OUT TIME MONTH 8									
	median	IQR	range						
	24	23 to 24	6 to 24						
1 to 8	3	0	3	0.00					
9 to 23	8	24	32	0.75					
24	13	39	52	0.75					
per hour					1.15	1.02	1.30	0.02	**0.02**
AVERAGE DAILY TURN OUT TIME MONTH 9									
	median	IQR	range						
	24	23 to 24	5 to 24						
1 to 8	4	2	6	0.33	0.19	0.03	1.18	0.08	
9 to 23	3	18	21	0.86	2.37	0.62	9.1	0.21	
24	17	43	60	0.72	**ref**				**0.07**
AVERAGE DAILY TURN OUT TIME MONTH 10									
	median	IQR	range						
	24	24 to 24	5 to 24						

1 to 8	3	3	6	0.50					
9 to 23	3	15	18	0.83					
24	17	43	60	0.72					
per hour					1.06	0.96	1.17	0.26	0.26
AVERAGE DAILY TURN OUT TIME MONTH 11									
	median	IQR	range						
	24	24 to 24	5 to 24						
1 to 8	2	2	4	0.50	0.50	0.06	4.4	0.53	
9 to 23	2	17	19	0.89	3.73	0.64	21.74	0.14	
24	17	36	53	0.68	**ref**				0.25
AVERAGE DAILY TURN OUT TIME MONTH 12									
	median	IQR	range						
	24	18 to 24	4 to 24						
1 to 8	2	3	5	0.60	1.35	0.13	13.78	0.80	
9 to 23	1	26	27	0.96	26.28	1.9	362.82	0.02	
24	17	24	41	0.58	**ref**				**0.05**
AVERAGE DAILY TURN OUT TIME MONTHS 1-3									
	median	IQR	range						
	9	6 to 11	2 to 17						
2 to 6	10	17	27	0.63	1.24	0.41	3.75	0.69	
7 to 11	4	22	26	0.84	4.03	1.01	15.08	0.04	
12 to 17	11	15	26	0.58	**ref**				**0.10**

AVERAGE DAILY TURN OUT TIME MONTHS 4-6									
	median	IQR	range						
	23	21 to 24	6 to 24						
1 to 8	1	0	1	0.00					
9 to 23	19	54	73	0.74					
24	6	11	17	0.65					
per hour					1.22	1.04	1.44	0.02	**0.02**
AVERAGE DAILY TURN OUT TIME MONTHS 7-9									
	median	IQR	range						
	24	23 to 24	24						
1 to 8	0								
9 to 23	12	40	52	0.77					
24	11	21	32	0.65					
per hour					1.18	0.99	1.41	0.06	**0.06**
AVERAGE DAILY TURN OUT TIME MONTHS 10-12									
	median	IQR	range						
	24	19 to 24	7 to 24						
1 to 8	1	1	2	0.50	0.94	0.03	28.82	0.97	
9 to 23	3	30	33	0.90	8.32	1.62	42.71	0.01	
24	15	20	35	0.57	**ref**				**0.03**
AVERAGE DAILY TURN OUT TIME MONTHS 1-6									
	median	IQR	range						

	17	15 to 18	5 to 23						
1 to 8	2	0	2	0.00					
9 to 23	24	65	89	0.73					
24	0								
per hour					1.20	1.03	1.40	0.02	**0.02**
AVERAGE DAILY TURN OUT TIME MONTHS 7-12									
	median	IQR	range						
	24	20 to 24	10 to 24						
1 to 8	0								
9 to 23	13	48	61	0.79					
24	10	13	23	0.56					
per hour					0.94	0.74	1.19	0.63	0.63
AVERAGE DAILY TURN OUT AREA MONTH 1 (acres)									
	median	IQR	range						
	1	0.5 to 1.5	0 to 5						
0 to 0.5	15	2	47	0.68					
0.5 to 1	6	29	35	0.83					
>1	9	31	40	0.78					
per acre					1.09	0.65	1.84	0.73	0.73
AVERAGE DAILY TURN OUT AREA MONTH 2									
	median	IQR	range						

	3	1 to 5	0.5 to 9						
0.5 to 1.5	9	20	29	0.69					
2 to 4.5	11	23	34	0.68					
>4.5	6	23	29	0.79					
per acre					1.13	0.92	1.39	0.25	0.25
AVERAGE DAILY TURN OUT AREA MONTH 3									
	median	IQR	range						
	5	3 to 7	0.5 to 9						
0.5 to 4	12	21	33	0.64					
4.5 to 7	5	26	31	0.84					
>7	10	18	28	0.63					
per acre					1.03	0.88	1.22	0.68	0.68
AVERAGE DAILY TURN OUT AREA MONTH 4									
	median	IQR	range						
	5	3 to 7	1 to 11						
0.5 to 4	10	22	32	0.68					
4.5 to 6.5	9	27	36	0.75					
>6.5	9	19	28	0.69					
per acre					0.99	0.83	1.19	0.92	0.92
AVERAGE DAILY TURN OUT AREA MONTH 5									
	median	IQR	range						
	6	4 to 7	0.5 to 13						

0.5 to 4.5	8	22	30	0.73						
5 to 7	8	24	32	0.75						
>7	12	21	33	0.64						
per acre					0.98	0.79	1.23	0.88	0.88
AVERAGE DAILY TURN OUT AREA MONTH 6										
	median	IQR	range							
	6	4 to 7	0.5 to 17							
0.5 to 5	9	21	30	0.70						
5.5 to 8	13	26	39	0.67						
>8	6	20	26	0.77						
per acre					1.11	0.98	1.26	0.11	**0.11**
AVERAGE DAILY TURN OUT AREA MONTH 7										
	median	IQR	range							
	7	4 to 8	0.5 to 17							
0.5 to 5	11	19	30	0.63						
5 to 8	9	23	32	0.72						
>8	6	22	28	0.78						
per acre					1.01	0.95	1.19	0.26	0.26
AVERAGE DAILY TURN OUT AREA MONTH 8										
	median	IQR	range							
	7	4 to 10	0.5 to 17							
0.5 to 5.5	9	20	29	0.69						

6 to 8.5	7	22	29	0.76					
>8.5	8	21	29	0.72					
per acre					1.05	0.94	1.17	0.36	0.36
AVERAGE DAILY TURN OUT AREA MONTH 9									
	median	IQR	range						
	7	4 to 10	0.5 to 17						
0.5 to 5	9	20	29	0.69					
5.5 to 8	5	24	29	0.82					
>8	10	19	29	0.65					
per acre					1.03	0.93	1.14	0.59	0.59
AVERAGE DAILY TURN OUT AREA MONTH 10									
	median	IQR	range						
	7	4 to 10	0.5 to 17						
0.5 to 5	8	20	28	0.71					
5.5 to 8	7	22	29	0.76					
>8	8	19	27	0.7					
per acre					1.05	0.94	1.16	0.39	0.39
AVERAGE DAILY TURN OUT AREA MONTH 11									
	median	IQR	range						
	7	4 to 10	0.5 to 17						
0.5 to 5	9	20	29	0.69					
5.5 to 9.5	7	22	29	0.76					
>9.5	8	21	29	0.72					

per acre					1.05	0.94	1.19	0.35	0.35
AVERAGE DAILY TURN OUT AREA MONTH 12									
	median	IQR	range						
	7	4 to 10	0.5 to 17						
0.5 to 5	9	17	26	0.65					
5.5 to 10.5	8	21	29	0.72					
>10.5	3	15	18	0.83					
per acre					1.04	0.92	1.19	0.48	0.48
AVERAGE DAILY TURN OUT AREA MONTH 1-3									
	median	IQR	range						
	3	1 to 5	0.5 to 6						
0.5 to 2	10	17	27	0.63					
2.5 to 4	7	19	26	0.73					
>4	8	18	26	0.69					
per acre					1.14	0.87	1.51	0.32	0.32
AVERAGE DAILY TURN OUT AREA MONTH 4-6									
	median	IQR	range						
	6	4 to 8	1 to 11						
1 to 4.5	10	18	28	0.64					
4.5 to 7	6	22	28	0.78					
>7	9	19	28	0.67					
per acre					1.06	0.94	1.18	0.34	0.34

AVERAGE DAILY TURN OUT AREA MONTH 7-9									
	median	IQR	range						
	7	4 to 10	0.5 to 17						
0.5 to 5	9	17	27	0.65					
5.5 to 8	8	21	29	0.72					
>8	3	15	18	0.83					
per acre					1.04	0.92	1.19	0.48	0.48
AVERAGE DAILY TURN OUT AREA MONTH 10-12									
	median	IQR	range						
	7	4 to 10	0.5 to 17						
0.5 to 5	9	17	27	0.65					
5.5 to 10	8	21	29	0.72					
>10	3	15	18	0.83					
per acre					1.04	0.92	1.19	0.48	0.48
AVERAGE DAILY TURN OUT AREA MONTH 1-6									
	median	IQR	range						
	5	3 to 6	0.5 to8						
0.5 to 4	9	22	31	0.71					
4.5 to 5.5	8	22	30	0.73					
>5.5	9	21	30	0.7					
per acre					1.13	0.87	1.45	0.35	0.35
AVERAGE DAILY TURN OUT AREA MONTH 7-12									

	median	IQR	range						
	7	5 to 10	0.5 to 17						
0.5 to 5	8	16	24	0.67					
5.5 to 9.5	5	18	23	0.78					
>9.5	6	17	23	0.74					
per acre					1.05	0.89	1.25	0.54	0.54
**EARLY-LIFE DISEASE/INJURY**									
DEVELOPMENTAL ORTHOPAEDIC DISEASE									
no	17	73	90	0.81	**ref**				**0.04**
yes	14	25	39	0.64	0.41	0.18	0.96	0.04	
MUSCULOSKELETAL INJURY									
no	26	75	101	0.74	**ref**				0.39
yes	5	23	28	0.82	1.62	0.54	4.79	0.39	
MISCELLANEOUS MUSCULOSKELETAL CONDITION									
no	29	95	124	0.77	**ref**				0.38
yes	2	3	5	0.6	0.42	0.06	2.89	0.38	
CONDITION AFFECTING THE FOOT									
no	27	88	115	0.76	**ref**				0.64
yes	4	10	14	0.71	0.73	0.19	2.76	0.64	
PNEUMONIA									
no	29	92	121	0.76	**ref**				0.94
yes	2	6	8	0.75	0.94	0.17	5.12	0.94	

COLIC									
no	28	96	124	0.77	**ref**				**0.08**
yes	3	2	5	0.40	0.18	0.03	1.22	0.08	
ENTERITIS/COLITIS									
no	28	93	121	0.77	**ref**				0.40
yes	3	5	8	0.62	0.52	0.11	2.38	0.40	
**MARE ATTRIBUTES**									
AGE									
	median	IQR	range						
	7	5 to 10	3 to 22						
3 to 5	7	25	32	0.78					
6 and 7	7	26	33	0.79					
8 to 10	8	25	33	0.76					
>10	9	22	31	0.71					
per year					0.96	0.86	1.01	0.45	0.45
NUMBER OF PREVIOUS FOALS									
	median	IQR	range						
	2	2 to 4	0 to 13						
0 (maiden)	6	20	26	0.77					
1	5	13	18	0.72					
2	5	18	23	0.78					
3 and 4	10	26	36	0.72					
>4	5	21	26	0.81					

per foal					1.02	0.87	1.2	0.78	0.78
STATUS									
Foaling	18	65	83	0.78	**ref**				0.64
Barren/Aborted	7	18	25	0.72	0.75	0.25	2.33	0.63	
Maiden	6	15	21	0.71	0.58	0.18	1.89	0.37	
**GESTATIONAL HEALTH/MEDICATION**									
ILLNESS/INJURY									
no	13	48	61	0.79	**ref**				0.99
yes	7	26	33	0.79	1.01	0.26	2.83	0.99	
MEDICATION									
no	12	39	51	0.76	**ref**				0.33
yes	7	2	45	0.84	1.67	0.59	4.69	0.33	
