# Supplementary material for: Born to run? Associations between gestational and early‐life exposures and later‐life performance outcomes in Thoroughbreds
Source: Equine Vet J. 2025 Aug 25;58(4):1071–81. doi: 10.1111/evj.70084 (PMC13244176; doi:10.1111/evj.70084)
Supplement: Supplementary file 4 — Table S2. The distribution of the total number of race starts made by the end of the fourth year of life by exposure and results of univariable logistic regression analysis to investigate associations between gestational and early‐life exposures and the total number of race starts made by the end of the fourth year of life, in a cohort of 84 flat‐bred Thoroughbreds born on six stud farms across the United Kingdom between 1 January 2019 and 31 December 2020. [file EVJ-58-1071-s003.pdf]

Equine Veterinary Journal BEVA

Table S2: The distribution of the total number of race starts made by the end of the fourth year of life by exposure and results of univariable logistic regression analysis to investigate associations between gestational and early-life exposures and the total number of race starts made by the end of the fourth year of life, in a cohort of 84 flat-bred Thoroughbreds born on six stud farms across the United Kingdom between 1 January 2019 and 31 December 2020.

***n* RUNS**										
	**DESCRIPTIVE STATISTICS (*n* runs)**					**UNIVARIABLE**				
**FOAL ATTRIBUTES**	***n***	**median**	**IQR**	**range**		**Coef.**	**95%CI**		**Wald P**	**P**
SEX										
Colt	44	7	4 to 11	0 to 22		**ref**				0.56
Filly	40	7	5 to 11	0 to 23		-0.61	-2.67	1.44	0.56	
MONTH OF BIRTH										
January	7	5	2 to 8	2 to 9		-3.20	-6.9	0.54	0.09	**0.17**
February	23	7	5 to 11	0 to 22		-0.40	-2.9	2.12	0.76	
March	31	8	5 to 11	0 to 23		**ref**				
April	19	5	2 to 8	0 to 16		-2.95	-5.67	-0.24	0.03	
May	4	8	4 to 11	3 to 13		-0.97	-5.85	3.91	0.70	
YEAR OF BIRTH										
2019	72	7	5 to 11	0 to 23		**ref**				0.98
2020	12	7	4 to 9	2 to 16		-0.03	3.21	3.16	0.98	
GESTATION LENGTH (days)										
314-337	29	7	4 to 11	0 to 23		**ref**				0.73
238-343	22	7	5 to 11	0 to 16		-0.31	-2.98	2.35	0.82	
344-351	19	5	2 to 10	0 to 18		-1.46	-4.27	1.35	0.31	
352-397	14	8	5 to 11	0 to 17		0.09	-3.02	3.21	0.95	
**EARLY-LIFE MANAGEMENT**										

AGE AT WEANING (days)										
112-155	16	5	2 to 9	0 to 13						
156-170	11	10	7 to 11	4 to 16						
171-179	14	8	7 to11	0 to 13						
180-250	15	7	3 to 12	0 to 23						
per day						0.06	0.02	0.11	0.005	**0.005**
SOLD AS A FOAL										
no	78	7	4 to 11	0 to 23		**ref**				0.64
yes	6	8	5 to 12	4 to 13		1.36	-2.77	5.48	0.64	
SOLD AS A YEARLING										
no	44	6	3 to 11	0 to 23		**ref**				0.24
yes	40	8	5 to 11	0 to 22		1.29	-0.88	3.46	0.24	
AGE FIRST TURNED OUT 24/7 (days)										
32 to 65	13	8	5 to 13	0 to16		**ref**				**0.16**
66 to 80	19	8	4 to 11	0 to 23		-0.37	-3.66	2.92	0.83	
81 to 98	17	6	3 to 7	2 to 11		-2.39	-5.8	1.02	0.17	
99 to 174	11	9	5 to 12	4 to 22		1.15	-2.2	5.22	0.42	
per day						0.99	0.98	1.01	0.45	0.45
AVERAGE DAILY TURN OUT TIME MONTH 1 (hours)										
0 to 2	25	5	3 to 11	0 to 23						
3 to 5	24	7	2 to 11	0 to 12						
6 to 7	33	8	5 to 11	0 to 18						
per hour						0.3	-0.38	0.99	0.38	0.38

AVERAGE DAILY TURN OUT TIME MONTH 2										
1 to 5	19	8	4 to 11	0 to 22						
6 to 7	14	8	5 to 11	0 to 13						
8 to 23	26	7	5 to 11	0 to 23						
per hour						0.06	-0.23	0.36	0.67	0.67
AVERAGE DAILY TURN OUT TIME MONTH 3										
1 to 8	17	7	4 to 10	2 to 22		0.96	0.35	2.67	0.94	
9 to 23	21	8	5 to 11	0 to 23		3.3	0.91	11.97	0.07	
24	17	8	5 to 12	0 to 16		**ref**				0.98
AVERAGE DAILY TURN OUT TIME MONTH 4										
1 to 8	4	7	4 to 12	4 to 16						
9 to 23	30	8	5 to 11	0 to 22						
24	23	8	3 to 11	0 to 23						
per hour						-0.09	-0.35	0.16	0.48	0.48
AVERAGE DAILY TURN OUT TIME MONTH 5										
1 to 8	0									
9 to 23	24	7	5 to 11	0 to 13						
24	32	8	4 to 11	0 to 12						
per hour						0.25	-0.59	1.11	0.55	0.55
AVERAGE DAILY TURN OUT TIME MONTH 6										
1 to 8	1	5	5	5						
9 to 23	23	7	5 to 11	0 to 13						
24	32	8	5 to 12	0 to 23						

per hour						0.11	-0.47	0.7	0.69	0.69
AVERAGE DAILY TURN OUT TIME MONTH 6										
1 to 8	0									
9 to 23	24	6	4 to 10	0 to 12						
24	30	10	5 to 12	0 to 23						
per hour						0.82	-0.18	1.82	0.11	**0.11**
AVERAGE DAILY TURN OUT TIME MONTH 8										
1 to 8	0									
9 to 23	18	8	5 to 11	0 to 13						
24	25	7	4 to 11	0 to 23						
per hour						-0.2	-0.83	0.42	0.53	0.53
AVERAGE DAILY TURN OUT TIME MONTH 9										
1 to 8	0									
9 to 23	13	11	7 to 11	5 to 13						
24	28	7	3 to 11	0 to 23						
per hour						-0.02	-0.32	0.28	0.90	0.90
AVERAGE DAILY TURN OUT TIME MONTH 10										
1 to 8	3	5	5 to 11	5 to 11						
9 to 23	14	8	5 to 11	1 to 12						
24	34	7	4 to 11	0 to 23						
per hour						0.04	-0.24	0.33	0.75	0.75

AVERAGE DAILY TURN OUT TIME MONTH 11										
1 to 8	2	6	5 to 8	5 to 8						
9 to 23	12	11	7 to 12	2 to 13						
24	32	7	3 to qq	0 to 23						
per hour						-0.04	-0.32	0.25	0.81	0.81
AVERAGE DAILY TURN OUT TIME MONTH 12										
1 to 8	2	5	5 to 5	5 to 5						
9 to 23	21	8	6 to 11	1 to 13						
24	21	7	5 to 11	0 to 23						
per hour						-0.05	-0.33	0.24	0.75	0.75
AVERAGE DAILY TURN OUT TIME MONTHS 1-3										
2 to 6	16	5	3 to 11	0 to 22						
7 to 11	19	8	5 to 11	1 to 23						
12 to 17	13	8	5 to 13	0 to 16						
per hour						0.03	-0.38	0.43	0.89	0.89
AVERAGE DAILY TURN OUT TIME MONTHS 4-6										
1 to 8	0	n/a	n/a	n/a						
9 to 23	44	7	5 to 11	0 to 22						
24	10	9	3 to 13	0 to 23						
per hour						-0.19	-0.93	0.55	0.61	0.61

AVERAGE DAILY TURN OUT TIME MONTHS 7-9										
1 to 8	0									
9 to 23	31	8	5 to 11	0 to 13						
24	20	7	3 to 11	0 to 23						
per hour						0.001	-0.64	0.64	0.99	0.99
AVERAGE DAILY TURN OUT TIME MONTHS 10-12										
1 to 8	1	5	5 to 5	5 to 5		0.94	0.03	28.82	0.97	
9 to 23	23	8	5 to 11	1 to 13		8.32	1.62	42.71	0.01	
24	18	7	5 to 12	0 to 23		**ref**				**0.03**
AVERAGE DAILY TURN OUT TIME MONTHS 1-6										
1 to 8	0									
9 to 23	54	8	5 to 11	0 to 23						
24	0	0	0	0						
per hour						-0.02	-0.48	0.47	0.99	0.99
AVERAGE DAILY TURN OUT TIME MONTHS 7-12										
1 to 8	0									
9 to 23	38	7	5 to 11	0 to 13						
24	13	10	5 to 13	0 to 23						
per hour						0.03	-0.51	0.58	0.63	0.63

AVERAGE DAILY TURN OUT AREA MONTH 1										
0 to 0.5	26	5	4 to 10	0 to 22						
0.5 to 1	27	6	2 to 11	0 to 23						
>1	29	8	5 to 11	0 to 17						
per acre						0.47	-0.69	1.64	0.43	0.43
AVERAGE DAILY TURN OUT AREA MONTH 2										
0.5 to 1.5	19	7	4 to 11	0 to 22						
2 to 4.5	21	7	5 to 11	1 to 23						
>4.5	19	8	5 to 11	0 to 16						
per acre						0.26	-0.28	0.8	0.34	0.34
AVERAGE DAILY TURN OUT AREA MONTH 3										
0.5 to 4	18	8	4 to 11	2 to 22						
4.5 to 7	21	8	4 to 11	0 to 23						
>7	16	7	5 to 11	0 to 13						
per acre						-0.06	-0.57	0.44	0.80	0.80
AVERAGE DAILY TURN OUT AREA MONTH 4										
0.5 to 4.5	19	7	5 to 11	1 to 13						
5 to 7	19	8	5 to 11	0 to 22						
>7	19	10	3 to 12	0 to 23						
per acre						0.08	-0.44	0.6	0.74	0.74

AVERAGE DAILY TURN OUT AREA MONTH 5										
0.5 to 4.5	17	7	5 to 11	2 to 13						
5 to 7	21	8	5 to 11	0 to 23						
>7	18	7	3 to 12	0 to 22						
per acre						-0.04	-0.56	0.47	0.87	0.87
AVERAGE DAILY TURN OUT AREA MONTH 6										
0.5 to 5	18	8	5 to 11	2 to 13						
5.5 to 8	21	8	5 to 12	0 to 22						
>8	17	7	3 to 11	0 to 23						
per acre						-0.11	-0.46	0.23	0.51	0.51
AVERAGE DAILY TURN OUT AREA MONTH 7										
0.5 to 5	17	8	5 to 11	2 to 12						
5 to 8	17	10	5 to 12	0 to 16						
>8	20	7	4 to 11	0 to 23						
per acre						-0.06	-0.36	0.24	0.69	0.69
AVERAGE DAILY TURN OUT AREA MONTH 8										
0.5 to 5.5	16	9	3 to 11	2 to 12						
6 to 8.5	19	8	4 to 12	0 to 23						
>8.5	18	7	5 to 11	0 to 22						
per acre						-0.10	-0.41	0.2	0.49	0.49

AVERAGE DAILY TURN OUT AREA MONTH 9										
0.5 to 5	17	8	4 to 11	2 to 12						
5.5 to 8	19	10	5 to 12	0 to 22						
>8	17	6	5 to 11	0 to 23						
per acre						-0.11	-0.41	0.17	0.44	0.44
AVERAGE DAILY TURN OUT AREA MONTH 10										
0.5 to 5	16	9	4 to 11	2 to 13						
5.5 to 8	19	8	5 to 12	0 to 22						
>8	16	6	4 to 10	0 to 23						
per acre						-0.71	-0.35	0.21	0.62	0.62
AVERAGE DAILY TURN OUT AREA MONTH 11										
0.5 to 5	15	9	7 to 11	3 to 13		**ref**				0.41
5.5 to 9.5	15	11	5 to 12	0 to 22		0.18	-0.32	3.67	0.91	
>9.5	16	5	2 to 10	0 to 23		-1.91	-5.35	1.51	0.27	
AVERAGE DAILY TURN OUT AREA MONTH 12										
0.5 to 5	14	7	5 to 11	2 to 12		**ref**				**0.08**
5.5 to 10.5	18	11	5 to 11	0 to 23		2.23	-1.06	5.52	0.18	
>10.5	12	5	3 to 10	0 to 11		-1.63	-5.27	2.01	0.38	
AVERAGE DAILY TURN OUT AREA MONTH 1-3										
0.5 to 2	16	8	4 to 10	0 to 22		**ref**				0.86
2.5 to 4	17	6	4 to 11	0 to 23		-0.28	-3.74	3.17	0.87	
>4	15	8	7 to 12	0 to 16		0.7	-2.93	3.34	0.7	

AVERAGE DAILY TURN OUT AREA MONTH 4-6										
1 to 4.5	15	5	4 to 10	2 to 13		**ref**				**0.19**
4.5 to 7	18	11	7 to 12	0 to 22		2.82	-0.44	6.09	0.09	
>7	19	7	3 to 11	0 to 23		0.58	-2.64	3.81	0.72	
AVERAGE DAILY TURN OUT AREA MONTH 7-9										
0.5 to 5	16	8	3 to 11	2 to 12						
5.5 to 8	16	8	7 to 12	0 to 13						
>8	19	6	3 to 11	0. to 23						
per acre						-0.10	-0.41	0.21	0.52	0.52
AVERAGE DAILY TURN OUT AREA MONTH 10-12										
0.5 to 5	14	8	5 to 11	2 to 13		**ref**				0.44
5.5 to 10	13	11	7 to 12	0 to 22		1.25	-2.47	4.98	0.51	
>10	15	6	3 to 11	0 to 23		-1.15	-4.74	2.44	0.53	
AVERAGE DAILY TURN OUT AREA MONTH 1-6										
0.5 to 4	19	5	4 to 10	2 to 22		**ref**				0.44
4.5 to 5.5	15	11	7 to 12	0 to 23		1.74	-1.5	4.98	0.29	
>5.5	20	7	4 to 11	0 to 13		-0.19	-3.2	2.81	0.90	
AVERAGE DAILY TURN OUT AREA MONTH 7-12										
0.5 to 5	13	8	5 to 11	2 to 12		**ref**				0.26
5.5 to 9.5	14	11	7 to 12	0 to 22		2.00	-1.67	5.68	0.28	
>9.5	16	6	2 to 11	0 to 23		-0.92	-4.54	2.69	0.62	
**EARLY-LIFE DISEASE/INJURY**										
DEVELOPMENTAL ORTHOPAEDIC DISEASE										

no	65	7	4 to 11	0 to 23		**ref**				0.44
yes	19	7	5 to 12	2 to 16		0.98	-1.52	3.48	0.44	
MUSCULOSKELETAL INJURY										
no	64	7	4 to 11	0 to 23		**ref**				0.90
yes	20	7	5 to 11	0 to 13		-0.16	-2.66	2.34	0.90	
MISCELLANEOUS MUSCULOSKELETAL CONDITION										
no	81	7	5 to 11	0 to 23		**ref**				0.76
yes	3	5	4 to 10	4 to 10		-0.87	-6.53	4.79	0.76	
CONDITION AFFECTING THE FOOT										
no	76	7	4 to 11	0 to 23		**ref**				0.79
yes	8	8	3 to 10	0 to 11		-0.5	-4.25	3.26	0.79	
PNEUMONIA										
no	80	7	4 to 11	0 to 23		**ref**				**0.11**
yes	4	11	8 to 14	5 to 16		3.9	-0.92	8.72	0.11	
COLIC										
no	83	7	5 to 11	0 to 23		**ref**				0.27
yes	1	2	2	2.00		-5.28	-14.74	4.17	0.27	
ENTERITIS/COLITIS										
no	80	7	4 to 11	0 to 23		**ref**				0.77
yes	4	7	3 to 10	2 to 11		-0.71	-5.56	4.13	0.77	
**MARE ATTRIBUTES**										
AGE										

3 to 5	23	7	5 to 11	1 to 18		**ref**				**0.03**
6 and 7	22	7	5 to 11	0 to 23		0.95	-1.75	3.64	0.49	
8 to 10	19	6	2 to 11	0 to 16		-3.15	-6.08	-0.22	0.03	
>10	20	8	4 to 11	0 to 13		-1.68	-4.47	1.11	0.24	
NUMBER OF PREVIOUS FOALS										
0 (maiden)	18	7	5 to 10	1 to 18		**ref**				0.60
1	10	10	4 to 11	0 to 13		-0.21	-3.87	3.43	0.91	
2	17	8	5 to 11	0 to 23		0.36	-2.91	3.62	0.83	
3 and 4	20	6	4 to 10	0 to 16		-1.71	-4.86	1.43	0.29	
>4	19	7	2 to 11	0 to 13		-1.45	-4.51	1.61	0.35	
STATUS										
Foaling	55	7	4 to 11	0 to 23		**ref**				0.80
Barren/Aborted	16	8	5 to 11	0 to 18		0.55	-2.18	3.28	0.69	
Maiden	13	7	5 to 7	1 to 17		-0.64	-3.60	2.31	0.67	
**GESTATIONAL HEALTH/MEDICATION**										
ILLNESS/INJURY										
no	42	8	5 to 11	0 to 17		**ref**				**0.16**
yes	23	5	3 to 11	0 to 23		-1.62	-3.9	0.66	0.16	
MEDICATION										
no	34	8	4 to 11	0 to 16		**ref**				0.50
yes	35	7	5 to 11	0 to 23		0.8	-1.51	3.12	0.50	
