# Supplementary material for: Born to run? Associations between gestational and early‐life exposures and later‐life performance outcomes in Thoroughbreds
Source: Equine Vet J. 2025 Aug 25;58(4):1071–81. doi: 10.1111/evj.70084 (PMC13244176; doi:10.1111/evj.70084)
Supplement: Supplementary file 5 — Table S3. The distribution of the total prizemoney won (GBP) by the end of the fourth year of life by exposure and results of univariable logistic regression analysis to investigate associations between gestational and early‐life exposures and the natural logarithm of the total prizemoney won (lnGBP) by the end of the fourth year of life, in a cohort of 84 flat‐bred Thoroughbreds born on six stud farms across the United Kingdom between 1 January 2019 and 31 December 2020. [file EVJ-58-1071-s004.pdf]

Equine Veterinary Journal BEVA

Table S3: The distribution of the total prizemoney won (GBP) by the end of the fourth year of life by exposure and results of univariable logistic regression analysis to investigate associations between gestational and early-life exposures and the natural logarithm of the total prizemoney won (lnGBP) by the end of the fourth year of life, in a cohort of 84 flat-bred Thoroughbreds born on six stud farms across the United Kingdom between 1 January 2019 and 31 December 2020.

**TOTAL PRIZEMONEY**									
	**DESCRIPTIVE STATISTICS (GBP)**				**UNIVARIABLE (outcome in natural log scale)**				
**FOAL ATTRIBUTES**	***n***	**median**	**IQR**	**range**	**Coef.**	**95%CI**		**Wald *P***	***P***
SEX									
Colt	44	7,950	3,294 to 27,013	0 to 19,7601	**ref**				**0.09**
Filly	40	6,341	1,179 to 14,881	0 to 92,004	-0.64	-1.39	0.11	0.09	
MONTH OF BIRTH									
January	7	4,320	602 to 7,007	0 to 24,541	-1.04	-2.47	0.37	0.15	0.59
February	23	8,419	1,712 to 18,431	336 to 92,000	-0.13	-1.09	0.83	0.79	
March	31	9,316	4,954 to 22,591	0 to 123,712	**ref**				
April	19	6,710	2,347 to 17,509	0 to 197,601	-0.08	-1.12	0.97	0.88	
May	4	3,127	1,445 to 6,329	1,179 to 8,114	-0.84	-2.64	0.96	0.36	
YEAR OF BIRTH									
2019	72	7,262	1,846 to 18,431	0 to 197,601	**ref**				**0.16**
2020	12	6,898	333 to 11,827	0 to 52,663	-0.75	-1.79	0.29	0.16	
GESTATION LENGTH (days)									
314-337	29	5,832	1,039 to 11,851	0 to 123,711					
238-343	22	6,990	2,579 to 17,119	0 to 51,979					
344-351	19	6,723	1,846 to 20,202	602 to 197,601					
352-397	14	18,767	6,40 to 289,875	203 to 52,663					

per day					0.04	0.01	0.08	0.02	**0.02**
**EARLY-LIFE MANAGEMENT**									
AGE AT WEANING (days)									
112-155	16	3,448	600 to 8,419	0 to 51,979	**ref**				**0.05**
156-170	11	9,218	5,327 to 16,252	203 to 123,711	1.19	-0.15	2.53	0.08	
171-179	14	8,255	6,822 to 28,821	1,042 to 197,601	1.65	0.37	2.93	0.01	
180-250	15	5,041	649 to 13,455	0 to 92,004	0.36	-0.89	1.62	0.56	
SOLD AS A FOAL									
no	78	6,898	1,779 to 18,201	0 to 197,601	**ref**				0.63
yes	6	4,846	1,648 to 13,663	300 to 45,164	-0.35	-1.79	1.08	0.63	
SOLD AS A YEARLING									
no	44	6,598	1,179 to13,456	0 to 197,601	**ref**				**0.12**
yes	40	7,7,85	3,496 to 26,575	0 to 123,711	0.59	-0.16	1.34	0.12	
AGE FIRST TURNED OUT 24/7 (days)									
32 to 65	13	7,304	4,020 to 12,254	1,710 to 52,664					
66 to 80	19	9,218	1,042 to 18,767	0 to 51,979					
81 to 98	17	6,285	1,179 to 20,202	336 to 197,601					
99 to 174	11	5,379	1,192 to 11,611	0 to 27,452					
per day					-0.02	-0.04	0.01	0.12	**0.12**
AVERAGE DAILY TURN OUT TIME MONTH 1 (hours)									
0 to 2	25	4,061	1,477 to 8,419	0 to 45,164					
3 to 5	24	7,112	1,274 to 26,575	0 to 123,711					
6 to 7	33	9,552	4,225 to 18,377	0 to 197,601					

per hour					0.19	-0.02	0.41	0.08	**0.08**
AVERAGE DAILY TURN OUT TIME MONTH 2									
1 to 5	19	4,850	1,192 to 12,092	0 to 27,452					
6 to 7	14	7,785	1,042 to 28,821	203 to 197,601					
8 to 23	26	7,468	4,020 to 15,826	336 to 123,711					
per hour					0.06	-0.04	0.17	0.22	0.22
AVERAGE DAILY TURN OUT TIME MONTH 3									
1 to 8	17	1,710	602 to 7,007	0 to 27,452					
9 to 23	21	9,633	1,512 to 26,671	203 to 197,601					
24	17	8,256	6,285 to 20,202	0 to 123,711					
per hour					0.07	0.01	0.14	0.04	**0.04**
AVERAGE DAILY TURN OUT TIME MONTH 4									
1 to 8	4	3,546	856 to 6,139	0 to 7,007					
9 to 23	30	7,785	1,274 to 18,767	300 to 197,601					
24	23	7,402	4,320 to 13,456	0 to 123,711					
per hour					0.08	-0.01	0.18	0.08	**0.08**
AVERAGE DAILY TURN OUT TIME MONTH 5									
1 to 8	0								
9 to 23	24	8,419	3,448 to 28,821	0 to 197,601					
24	32	6,897	1,274 to 12,091	0 to 52,664					
per hour					0.09	-0.23	0.41	0.59	0.59
AVERAGE DAILY TURN OUT TIME MONTH 6									

1 to 8	1	649	649	649					
9 to 23	23	8.102	1,845 to 20,202	0 to 92,004					
24	32	6,776	1,712 to 13,456	0 to 197,601					
per hour					0.13	-0.07	0.34	0.19	**0.19**
AVERAGE DAILY TURN OUT TIME MONTH 6									
1 to 8	0								
9 to 23	24	6,730	1,042 to 18,342	300 to 197,601					
24	30	7,188	4,623 to 12,533	0 to 123,711					
per hour					0.20	-0.18	0.58	0.30	0.3
AVERAGE DAILY TURN OUT TIME MONTH 8									
1 to 8	0								
9 to 23	18	6,737	1,846 to 18,767	600 to 197,601					
24	25	7,007	1,710 to 12,456	0 to 123,711					
per hour					0.02	-0.21	0.26	0.87	0.87
AVERAGE DAILY TURN OUT TIME MONTH 9									
1 to 8	0								
9 to 23	13	7,785	4,730 to 26,575	1,042 to 92,004					
24	28	7,007	1,274 to 12,146	0 to 197,601					
per hour					0.07	-0.04	0.18	0.24	0.24
AVERAGE DAILY TURN OUT TIME MONTH 10									
1 to 8	3	1,712	649 to 4,703	649 to 4,703					
9 to 23	14	16,56	3,448 to 28,821	300 to 197,601					

24	34	7,007	1,710 to 11,611	0 to 123,711					
per hour					0.07	-0.03	0.18	0.16	**0.16**
AVERAGE DAILY TURN OUT TIME MONTH 11									
1 to 8	2	3,694	649 to 6,740	649 to 6740	**ref**				**0.08**
9 to 23	12	16,824	6,052 to 28,172	1,042 to 197,601	1.92	-0.45	4.29	0.11	
24	32	6,729	1,710 to 11,611	300 to 123,711	0.81	-1.45	3.09	0.47	
AVERAGE DAILY TURN OUT TIME MONTH 12									
1 to 8	2	845	649 to 1,042	649 to 1,042	**ref**				**0.06**
9 to 23	21	13,146	5,327 to 26,575	300 to197,601	2.49	0.23	4.76	0.03	
24	21	7,422	1,710 to 11,611	336 to 123,711	1.82	-0.45	4.11	0.11	
AVERAGE DAILY TURN OUT TIME MONTHS 1-3									
2 to 6	16	4,320	463 to 11,6110	0 to 29,770					
7 to 11	19	6,973	1,179 to 20,202	203 to 197,601					
12 to 17	13	8,114	6,285 to 12,146	3,448 to 52,664					
per hour					0.15	0.01	0.29	0.04	**0.04**
AVERAGE DAILY TURN OUT TIME MONTHS 4-6									
1 to 8	0								
9 to 23	44	6,973	1,274 to 18,767	0 to 197,601					
24	10	7,468	3,127 to 9,552	336 to 13,456					
per hour					0.28	0.01	0.56	0.04	**0.04**

AVERAGE DAILY TURN OUT TIME MONTHS 7-9									
1 to 8	0								
9 to 23	31	6,874	1,710 to 18,767	300 to 197,601					
24	20	7,758	4,543 to 11,611	0 to 123,711					
per hour					0.16	-0.08	0.40	0.18	**0.18**
AVERAGE DAILY TURN OUT TIME MONTHS 10-12									
1 to 8	1	649	649	649.00	**ref**				0.25
9 to 23	23	8,420	4,320 to 26,575	300 to 197,601	2.60	-0.63	5.84	0.11	
24	18	8,256	1,846 to 12.092	336 to 123,711	2.24	-1.03	5.52	0.17	
AVERAGE DAILY TURN OUT TIME MONTHS 1-6									
1 to 8	0								
9 to 23	54	6,973	1,710 to 14,881	0 to 197,601					
24	0								
per hour					0.21	-0.01	0.44	0.06	**0.06**
AVERAGE DAILY TURN OUT TIME MONTHS 7-12									
1 to 8	0								
9 to 23	38	6,740	1,710 to 18,431	0 to 197,601					
24	13	9,218	4,543 to 13,456	336 to 123,711					
per hour					0.09	-0.09	0.26	0.34	0.34
AVERAGE DAILY TURN OUT AREA MONTH 1 (acres)									
0 to 0.5	26	3.294	965 to 9,309	0 to 51,979					

0.5 to 1	27	6,025	891to 10,033	0 to 123,711					
>1	29	16,239	6,781 to 26,681	3,448 to 197,601					
per acre					0.69	0.31	1.07	0.001	**0.001**
AVERAGE DAILY TURN OUT AREA MONTH 2									
0.5 to 1.5	19	1,477	600 to 8,420	0 to 27,452					
2 to 4.5	21	5,327	1,710 to 12,092	301 to 123,711					
>4.5	19	16,252	7,785 to 22,591	4,320 to 197,601					
per acre					0.32	0.16	0.49	0.002	**0.002**
AVERAGE DAILY TURN OUT AREA MONTH 3									
0.5 to 4	18	3,546	463 to 11,611	0 to 51,979					
4.5 to 7	21	6,740	1,274 to 9,218	301 to 197,601					
>7	16	13,146	4,543 to 18,821	600 to 123,711					
per day					0.25	0.08	0.43	0.005	**0.005**
AVERAGE DAILY TURN OUT AREA MONTH 4									
0.5 to 4.5	19	6,728	463 to 11,611	0 to 92,004					
5 to 7	19	6,856	1,846 to 28,821	0 to 197,601					
>7	19	8,256	4,543 to 13,146	336 to 123,711					
per acre					0.17	0.02	0.36	0.08	**0.08**
AVERAGE DAILY TURN OUT AREA MONTH 5									
0.5 to 4.5	17	6,730	649 to 7,785	0 to 45,836					

5 to 7	21	9,550	2,647 to 28,172	0 to 197,601					
>7	18	8,338	5,414 to 15,578	301 to 123,711					
per acre					0.11	-0.08	0.30	0.25	0.25
AVERAGE DAILY TURN OUT AREA MONTH 6									
0.5 to 5	18	6,150	1,042 to 13,146	0 to 92,004					
5.5 to 8	21	8,114	4,543 to 26,575	0 to 197,601					
>8	17	7,492	1,445 to 12,774	301 to 123,711					
per acre					0.01	-0.11	0.13	0.82	0.82
AVERAGE DAILY TURN OUT AREA MONTH 7									
0.5 to 5	17	7,402	1,179 to 26,575	0 to 197,601	**ref**				0.40
5 to 8	17	5,041	1,492 to 8,185	0 to 29,770	-0.08	-2.04	0.44	0.20	
>8	20	7.575	3,448 to 12,456	301 to 123,711	-0.19	-1.29	1.01	0.75	
AVERAGE DAILY TURN OUT AREA MONTH 8									
0.5 to 5.5	16	7,205	2,750 to 22,503	0 to 51,979	**ref**				0.80
6 to 8.5	19	5,379	1,042 to 14,881	0 to 197,601	-0.35	-1.16	0.92	0.58	
>8.5	18	6,740	4,543 to 9,218	301 to 123,711	0.03	-1.24	1.29	0.72	
AVERAGE DAILY TURN OUT AREA MONTH 9									
0.5 to 5	17	6,973	1,712 to 18,431	0 to 197,601					
5.5 to 8	19	7,593	1,042 to 26,575	0 to 92,004					
>8	17	6,730	1,846 to 12,092	301 to 123,711					
per acre					-0.01	-0.11	-0.10	-0.93	0.93

AVERAGE DAILY TURN OUT AREA MONTH 10									
0.5 to 5	16	6,990	1,445 to 16,674	0 to 45,836					
5.5 to 8	19	9,480	1,710 to 27,452	203 to 123,711					
>8	16	6,508	1,846 to 9,218	336 to 197,601					
per acre					0.01	-0.10	0.11	0.91	0.91
AVERAGE DAILY TURN OUT AREA MONTH 11									
0.5 to 5	15	7,007	1,710 to 12,431	300 to 92,004	**ref**				**0.07**
5.5 to 9.5	15	19,861	4,320 to 29,770	463 to 197,601	0.80	-0.35	1.95	0.16	
>9.5	16	5,806	1,274 to 8,419	301 to 13,456	-0.57	-1.72	0.58	0.32	
AVERAGE DAILY TURN OUT AREA MONTH 12									
0.5 to 5	14	9,175	3,448 to 20,202	300 to 123,711	**ref**				**0.13**
5.5 to 10.5	18	11,997	6,164 to 27,013	336 to 197,601	0.27	-0.87	1.41	0.63	
>10.5	12	5,327	1,274 to 8,419	301 to 12,092	-0.95	-2.21	0.31	0.13	
AVERAGE DAILY TURN OUT AREA MONTH 1-3									
0.5 to 2	16	1,192	463 to 7,007	0 to 27,452					
2.5 to 4	17	6,629	1,560 to 10,655	301 to 45,836					
>4	15	17,342	7,785 to 29,770	4,320 to 197,601					
per acre					0.53	0.27	0.78	<0.001	**<0.001**
AVERAGE DAILY TURN OUT AREA MONTH 4-6									
1 to 4.5	15	5,327	463 to 7,401	0 to 45,836	**ref**				**0.04**
4.5 to 7	18	14,881	5,379 to 27,452	0 to 197,601	1.54	0.25	2.73	0.01	

>7	19	6,822	1,710 to 9,218	301 to 123,711	0.66	-0.52	1.85	0.26	
AVERAGE DAILY TURN OUT AREA MONTH 7-9									
0.5 to 5	16	6,990	1,111 to 15,789	0 to 51,979	**ref**				0.73
5.5 to 8	16	8,114	1,712 to 28,821	203 to 197,610	0.47	-0.79	1.74	0.45	
>8	19	6,730	1,846 to 12,092	301 to 123,711	0.09	-1.13	1.31	0.88	
AVERAGE DAILY TURN OUT AREA MONTH 10-12									
0.5 to 5	14	7,262	3,448 to 18,767	300 to 92,004	**ref**				**0.09**
5.5 to 10	13	22,503	9,481 to 40,400	301 to 197,601	0.87	-0.34	0.34	0.15	
>10	15	6,285	1,846 to 8,419	0 to 13,456	-0.50	-1.69	0.69	0.40	
AVERAGE DAILY TURN OUT AREA MONTH 1-6									
0.5 to 4	19	3,488	463 to 7,007	0 to 45,836					
4.5 to 5.5	15	14,881	7,785 to 28,821	336 to 197,601					
>5.5	20	8,114	4,320 to 13,146	301 to 123,711					
per day					0.33	0.04	0.63	0.03	**0.03**
AVERAGE DAILY TURN OUT AREA MONTH 7-12									
0.5 to 5	13	6,740	3,48 to 14,881	300 to 92,004	**ref**				**0.06**
5.5 to 9.5	14	20,202	8,114 to 28,821	336 to 197,601	0.98	-0.21	2.18	0.10	
>9.5	16	6,285	1,846 to 8,419	301 to 12,456	-0.42	-1.62	0.77	0.48	
**EARLY-LIFE DISEASE/INJURY**									
DEVELOPMENTAL ORTHOPAEDIC DISEASE									
no	65	8,256	3803 to 24,541	0 to 197,601	**ref**				**0.01**
yes	19	1,846	1,192 to 8,114	0 to 16,252	-1.15	-2.00	-0.30	0.01	

MUSCULOSKELETAL INJURY									
no	64	6,898	1,809 to 17,119	0 to 92,004	**ref**				0.65
yes	20	7,262	1,712 to 20,202	300 to 197,601	0.20	-0.70	1.11	0.65	
MISCELLANEOUS MUSCULOSKELETAL CONDITION									
no	81	7,007	1,908 to 18,431	0 to 197,601	**ref**				**0.05**
yes	3	1,274	300 to 1,710	300 to 1,710	-1.94	-3.88	-0.01	0.05	
CONDITION AFFECTING THE FOOT									
no	76	6,973	1,846 to 18,767	0 to 197,601	**ref**				0.39
yes	8	5,327	1,710 to 9,218	301 to 12,091	-0.58	-1.91	0.75	0.39	
PNEUMONIA									
no	80	6,990	1,711 to 18,431	0 to 197,601	**ref**				0.65
yes	4	5,041	2,652 to 10,130	600 to 14,881	-0.39	-2.13	1.34	0.65	
COLIC									
no	83	6,973	1,712 to 17,987	0 to 197,601	**ref**				0.95
yes	1	6,285	6,285	6,285	0.11	-3.29	3.51	0.95	
ENTERITIS/COLITIS									
no	80	7,205	1,908 to 18,431	0 to 197,601	**ref**				**0.05**
yes	4	896	531 to 2,948	463 to 4,703	-1.70	-3.40	0.01	0.05	
**MARE ATTRIBUTES**									
AGE									
3 to 5	23	6,973	1,846 to 15,968	463 to 45,836	**ref**				0.79
6 and 7	22	8,114	2,786 to 22,591	300 to 123,711	0.30	-0.72	1.33	0.56	

8 to 10	19	6,554	1,186 to 14,172	0 to 197,601	-0.25	-1.36	0.85	0.65	
>10	20	7,262	1,710 to 24,541	0 to 92,004	-0.05	-1.12	1.02	0.92	
NUMBER OF PREVIOUS FOALS									
0 (maiden)	18	6,869	1,846 to 16,227	463 to 45,836	**ref**				**0.13**
1	10	15,472	8,114 to 26,575	2,786 to 29,875	0.90	-0.45	2.24	0.19	
2	17	6,740	1,908 to 20,202	1,179 to 197,601	0.32	-0.82	1.48	0.57	
3 and 4	20	5,832	1,648 to 16,353	300 to 123,711	0.25	-0.94	1.25	0.79	
>4	19	5,100	301 to 12,092	0 to 92,004	-0.77	-1.87	0.33	0.17	
STATUS									
Foaling	55	6,781	1,712 to 15,472	0 to 197,601	**ref**				0.38
Barren/Aborted	16	15,968	2,786 to 29,875	203 to 52,664	0.52	-0.47	1.51	0.30	
Maiden	13	4,594	1,477 to 9,218	463 to 28,821	-0.35	-1.39	0.69	0.26	
**GESTATIONAL HEALTH/MEDICATION**									
ILLNESS/INJURY									
no	42	7,402	3,448 to 20,202	0 to 197,601	**ref**				0.41
yes	23	4,954	1,710 to 11,611	300 to 123,711	-0.38	-1.29	0.53	0.41	
MEDICATION									
no	34	6,822	1,477 to 13,146	0 to 197,601	**ref**				**0.17**
yes	35	8,420	2,786 to 24,541	300 to 123,711	0.59	-0.25	1.39	0.17	
